# Acidic microenvironment plays a key role in human melanoma progression through a sustained exosome mediated transfer of clinically relevant metastatic molecules

**DOI:** 10.1186/s13046-018-0915-z

**Published:** 2018-10-05

**Authors:** Zaira Boussadia, Jessica Lamberti, Fabrizio Mattei, Elisabetta Pizzi, Rossella Puglisi, Cristiana Zanetti, Luca Pasquini, Federica Fratini, Luca Fantozzi, Federica Felicetti, Katia Fecchi, Carla Raggi, Massimo Sanchez, Stefania D’Atri, Alessandra Carè, Massimo Sargiacomo, Isabella Parolini

**Affiliations:** 1 Global Health Center, Istituto Superiore di Sanità, Rome, Italy; 2 Oncology and Molecular Medicine Department, Istituto Superiore di Sanità, Rome, Italy; 3 Major Equipments and Core Facilities, Istituto Superiore di Sanità, Rome, Italy; 4 National Center for the Control and Evaluation of Medicine, Istituto Superiore di Sanità, Rome, Italy; 5 Center for Gender- specific Medicine, Istituto Superiore di Sanità, Istituto Superiore di Sanità, Rome, Italy; 6 Laboratory of Molecular Oncology, Istituto Dermopatico dell’Immacolata- IRCCS, Rome, Italy

**Keywords:** Exosomes, Melanoma progression, Tumor stage, Microenvironmental acidic pH

## Abstract

**Background:**

Microenvironment cues involved in melanoma progression are largely unknown. Melanoma is highly influenced in its aggressive phenotype by the changes it determinates in its microenvironment, such as pH decrease, in turn influencing cancer cell invasiveness, progression and tissue remodelling through an abundant secretion of exosomes, dictating cancer strategy to the whole host. A role of exosomes in driving melanoma progression under microenvironmental acidity was never described.

**Methods:**

We studied four differently staged human melanoma lines, reflecting melanoma progression, under microenvironmental acidic pHs pressure ranging between pH 6.0–6.7. To estimate exosome secretion as a function of tumor stage and environmental pH, we applied a technique to generate native fluorescent exosomes characterized by vesicles integrity, size, density, markers expression, and quantifiable by direct FACS analysis.

Functional roles of exosomes were tested in migration and invasion tests. Then we performed a comparative proteomic analysis of acid versus control exosomes to elucidate a specific signature involved in melanoma progression.

**Results:**

We found that metastatic melanoma secretes a higher exosome amount than primary melanoma, and that acidic pH increases exosome secretion when melanoma is in an intermediate stage, i.e. metastatic non-invasive.

We were thus able to show that acidic pH influences the intercellular cross-talk mediated by exosomes. In fact when exposed to exosomes produced in an acidic medium, pH *naïve* melanoma cells acquire migratory and invasive capacities likely due to transfer of metastatic exosomal proteins, favoring cell motility and angiogenesis.

A Prognoscan-based meta-analysis study of proteins enriched in acidic exosomes, identified 11 genes (HRAS, GANAB, CFL2, HSP90B1, HSP90AB1, GSN, HSPA1L, NRAS, HSPA5, TIMP3, HYOU1), significantly correlating with poor prognosis, whose high expression was in part confirmed in bioptic samples of lymph node metastases.

**Conclusions:**

A crucial step of melanoma progression does occur at melanoma intermediate –stage, when extracellular acidic pH induces an abundant release and intra-tumoral uptake of exosomes. Such exosomes are endowed with pro-invasive molecules of clinical relevance, which may provide a signature of melanoma advancement.

**Electronic supplementary material:**

The online version of this article (10.1186/s13046-018-0915-z) contains supplementary material, which is available to authorized users.

## Background

In melanoma cell systems, progression of tumorigenesis is influenced by changes of microenvironmental conditions in pre-malignant lesions. They involve complex interactions with surrounding structural endothelial cells, which ultimately lead to hypoxia and extracellular acidity.

Microenvironmental acidification is the consequence of an upregulated glycolysis which results in acute and chronic pH decrease of the local microenvironment, that in human melanoma has been reported to be about 6.4–7.3 [1, 2].

In the early phases of tumor progression, extracellular acidity influences gene expression by up modulation of several hundred genes encoding receptors, signal proteins, transcription factors, cytokines [3], involved in invasion, tissue remodeling, cell cycle control and proliferation [4, 5], thus leading to a more malignant cell phenotype.

However, microenvironment cues involved in the early steps of melanoma progression are largely unknown. Exosomes, 50–100 nm vesicles abundantly released into the extracellular space [6], represent an important source of information with respect to the pH variations in melanoma. In fact, it has been established that exosomes mirror the signaling molecules content of cell from which they are generated, and are able to shuttle in an autocrine or paracrine way their molecular content from donor to recipient cells [7]. Exosomes can also use distal endocrine modality to interfere with phenotype and cellular processes of target cells, playing multiple roles in tumor progression including enhanced immunosuppression [8], angiogenesis [9], and metastasis by conditioning bone marrow and pre metastatic niches [10–12].

A recent study reported that conditioned medium by acidic melanoma cells promoted invasiveness and lung metastasis development of non acidic tumor cell [13]. However, a role of exosomes secreted under microenvironment acidic pressure in promoting melanoma progression was not described so far.

Tumor cells constitutively release in the microenvironment a plethora of extracellular vesicles (EV) other than exosomes, (i.e. microparticles, oncosomes) of plasma membrane origin with totally different roles [14], that are co-isolated in all the most common procedures including ultracentrifugation. In fact, as recently reported, vesicles obtained by ultracentrifugation are endowed with markers of plasma membrane or endosome origin [15, 16].

Therefore, the lack of an elective procedure to monitor only the traffic of the entire exosome population has interfered so far with clear assessment of exosomes involvement in biological processes. In fact, also the exosome antigen-bead isolation and intracellular visualization methods (CD63, CD81, CD9) allowed the study of sub populations, without taking into account vesicles heterogeneity and consequent limited role in biological processes observed in vitro [17].

In this paper we used a procedure based on the generation of metabolically labelled 100 nm -sized vesicles of endosomal origin [18]. This was made possible by adding in cell cultures a fatty acid analogue (Bodipy FL -C_16_), that upon uptake enters in cellular lipid metabolism ultimately producing fluorescent exosomes (C_16_-exo). After isolation from culture supernatants, these exosomes can be rapidly examined and quantified by direct FACS analysis. Importantly, this method represents an improvement on exosomes FACS-based studies so far reported, and provides a quantitative correlation between exosome uptake and cell functional effects. By means of this procedure we elucidated a role of microenvironment acidity in melanoma progression. We evaluated exosome release under acidic pH pressure from four differently staged human melanoma cell lines, representative of various steps of melanoma development.

The extracellular pH in the central region of tumors decreases at pH 6.7 and below because of lactate accumulation [1, 19, 20]. In order to mimic this process in vitro, we lowered the pH in cell culture media to pH 6.7 or 6.0, and evaluated the exosome population by FACS quantification, markers expression and density evaluation.

Next, we set up in vitro functional studies on acid pH-responsive metastatic non invasive (MNI) cell line, and performed a comparative proteomic characterization of exosomes obtained from standard and acid cell culture. The proteome expression in acid condition indicated an upregulation of several proteins belonging to functional categories previously described in melanoma progression such as proteoglycans, focal adhesion and protein processing in endoplasmic reticulum [21–23].

To find a clinical relevance of our proteomic studies, we examined human epidemiological data using the PrognoScan database [24], a large collection of publicly available cancer microarray datasets with clinical correlation in melanoma patients. The resulting data was finally validated by IHC on bioptic samples.

## Methods

### Cell lines

We analyzed some human melanoma cell lines representative of various degree of malignancy. Specifically we utilized WM115 (PI) and WM266–4 (MI) commercially available, that upon arrival were expanded, freezed and stored under liquid nitrogen. Me1007 (EP) and Mel501 (MNI) were obtained by Istituto Nazionale Tumori (Milan, Italy), authenticated according to a standard short tandem repeat-based genotyping at Ospedale Policlinico San Martino (Genova, Italy), and periodically tested for mycoplasma contamination.

Mel501 represents a primary low invasive melanoma and Me1007 an early primary melanoma. WM115 is a primary invasive and WM266–4 an advanced metastatic melanoma obtained from the same patient. Me1007 and Mel501 carry no classical mutations, whereas the other cell lines are B-RAF mutated (V600D) (Additional file 1). Melanoma cells were cultured in RPMI-1640 or Dulbecco modified Eagle’s medium (DMEM) (GIBCO by Life Technologies) supplemented with 10% fetal calf serum (FCS), in a humidified 5% CO_2_ incubator.

The bioptic melanoma specimens used in this study were obtained from the archives of the Istituto Dermopatico dell’Immacolata-IRCCS (Rome-Italy). Signed informed consent was obtained from patients. For each patient, melanoma samples (primary and autologous metastasis) were analyzed. Sampling and handling of human tissue material were carried out in accordance with the ethical principle of the Declaration of Helsinki.

### Cell labelling with BodipyFL-C_16_, and acidic treatments

MNI and EP cells (50% confluence) were incubated with Bodipy FL -C_16_ (4,4-difluoro-5,7-dimethyl-4-bora-3a,4a-diaza-s-indacene-3-hexadecanoic acid) (C_16_) (Life Technologies) by addition of 7 μM C_16_ in medium supplemented with 0.3% FCS for 4 h at 37 °C, as previously described [18].

The culture media at various pHs were obtained as follows. For pH 6.7 condition, we minimized the pH change during the cell culture by addition of 20 mM MOPS (3-(N-morpholino) propanesulfonic acid) to medium containing FBS, then adjusted with 1 N HCL to pH 6.7. A stronger acid condition (pH 6.0) was obtained by the addition of 1 N HCl to medium containing FBS. After 24 h incubation pH was evaluated by pH -meter and found unchanged for pH 6.7 condition, whereas in pH 6.0 samples, pH value ranged between 6.1–6.3.

### Isolation of C_16_-exo

C_16_-exo recovery was performed as previously described [7, 18] by ultracentrifugation method with minor modifications (Additional file 2).

### Western blot

Western blot analysis was performed according to standard procedures. C_16_-exo and melanoma cells were resuspended in Laemmli sample buffer with freshly added 50 μM DTT. Antibodies listed below were used in accordance to the manufacturer’s instructions: Mouse monoclonal antibody to Alix (3A9 #MA183977, Thermo Scientific, Waltham, MA USA); Mouse monoclonal antibody to TSG101 (4A10 #GTX70255, GeneTex, Irvine, CA USA); Mouse monoclonal antibody to Calnexin (37/Calnexin #610524, BD Transduction Laboratories, Lexington, KY USA), Mouse monoclonal antibody to CD81 (B11 #sc166029, Santa Cruz Biotechnology, Dallas, TX USA), Mouse monoclonal antibody to Flotillin-1 (18/flotillin-1 #610821 BD Transduction Laboratories, San Josè, CA USA), Mouse monoclonal antibody to Hsp70 (3A3 #NB6001469, Novus Biologicals, Littleton, CO USA), Rabbit polyclonal antibody to HSP90α/β (H114 #sc7947, Santa Cruz Biotechnology, Dallas, TX USA), Rabbit polyclonal antibody to Cofilin (#3312, Cell Signaling Thechnology, Leiden, The Netherlands), Goat polyclonal antibody to Gelsolin (N-18 #sc6406, Santa Cruz Biotechnology, Dallas, TX USA).

### TLC analysis of fluorescent phospholipids

Fluorescent phospholipids were analyzed as described (18). Briefly, total lipids were extracted according to Folch procedure and analyzed by TLC. As solvent mixture we used chloroform/methanol/32% ammonia (65:35:5, *v*/v) [25]. For plate scanning see Additional file 3.

### FACS analysis and quantification of C_16_-exo

The presence of discrete and intact fluorescent vesicles in C_16_-exo pellet isolated by ultracentrifugation (Additional file 2) was assessed by FACS analysis as reported [18] (see Additional file 3).

### Optiprep™ gradient centrifugation

We used a discontinuous iodixanol gradient floatation as described [26] (Additional file 3). Refractive index of each fraction was assessed with a refractometer (Carl Zeiss) and the relative density was calculated using the linear relationship between refractive index (η) and the density (ρ) ρ = Aη- B, [27] . For FACS quantification, 2 μl of each fraction were resuspended with 200 μl PBS and analysed as above described.

### Mass spectrometry analysis and data processing

Exosomes (8 μg) obtained from ctr or pH 6.0-treated MNI cells incubated for 24 h in absence of FCS, were separated on precast 4–12% Bis-Tris Gels (Invitrogen) and stained with Coomassie Colloidal Blue. Each lane was cut into sequential 25 slices and treated as in [28] (Additional file 3).

Three independent experiments were performed. We identified in exo (pH 6.0) 212, 211 and 217 proteins, and in exo (ctr) 194, 239 and 130 proteins. Only proteins identified with two peptides in at least two experiments were considered, and their abundances were estimated by the normalized emPAI_norm_ values according to emPAI_norm_ = emPAI/Σ_i_ emPAI; where *i* = 1,N and N is the number of proteins [29]. Reproducibility assessments were carried out in both cases (pH 6.0 and control), and the two best correlated experiments (Pearson’s correlation higher than 0.8) were considered to calculate the averaged emPAI_norm_ value (186 in pH 6.0 and 157 in the control) (Additional file 3).

The emPAI_norm_ ratio (ρ) between emPAI_norm_ values of proteins identified in pH 6.0 and those identified in the control was calculated. According with the emPAI ratio proteins were classified as up-regulated in pH 6.0 (ρ ≥ 1.5), equally regulated in pH 6.0 and in the control (0.5 ≤ ρ ≤ 1.5) and down-regulated in pH 6.0 condition (ρ ≤ 0.5).

Functional analysis was performed by using the DAVID Bioinformatics Resources 6.8 [30]. Proteins up-, equally and down-regulated were mapped on KEGG pathways separately and over-represented categories (*P* ≤ 10^− 2^) were considered.

### C_16_-exo transfer and functional assays

To evaluate C_16_-exo transfer to target cells, increasing amounts (40 to 700 exosome per cell) of C_16_-exo (ctr), C_16_-exo (pH 6.0) were added to MNI cells in a 96 well plate in duplicate in 100 μl RPMI without serum and kept for 2 h at 37 °C in incubator. Then medium was removed, cells PBS-washed, detached and subjected to FC analysis as described [18]. Briefly, fluorescence data of C_16_-exo, cell samples, and Quantum^TM^ FITC-5 MESF (Bangs Laboratories, Inc.) standard curve were acquired and transformed into MESF (Molecules of Equivalent Soluble Fluorophores) using the QuickCal analysis template provided with each Quantum^TM^ MESF lot. Then MESF associated to cells were converted in number of transferred exosomes according the formula: (cell fluorescence (MESF)-cell autofluorescence (MESF))/ exosome fluorescence (MESF).

Migration and invasion studies were performed according to standard procedures.

Cell migration was assayed, as previously described [31], using uncoated cell culture inserts (Corning Costar Corporation, Cambridge, MA). Assays were incubated at 37 °C in 5% CO_2_, and after 72 h migration was evaluated by a colorimetric assay [32] at 620 nm in a microplate reader (Victor X3, Perkin Elmer). Invasion was evaluated as for chemotaxis on culture inserts previously coated with matrigel.

Cell viability was measured by exclusion of dead cells with Trypan Blue Solution (0.4%).

### Confocal microscopy

For Confocal Laser Scanner Microscopy (CLSM) analysis, cells were grown on sterilized coverslips for 24 h. Cells were metabolically labeled with C_16_ or treated with C_16_-exo**,** then fixed with paraformaldehyde (3%) (30 min, 4 °C), quenched with 10 mM NH_4_Cl and mounted on the microscope slide with Vectashield antifade mounting medium containing DAPI (Vector Laboratories, Burlingame, CA). For images captures see Additional file 3.

### Meta-analysis of the prognostic value of gene expression by PrognoScan

PrognoScan is a comprehensive online platform for evaluating potential tumor biomarkers and therapeutic targets [33].

Based on a large collection of cancer microarray datasets with clinical annotation on GEO databases [34], PrognoScan is a tool to assess the association between specific gene expression and prognosis in patients with cancer [24].

We used this online database to validate metastasis-related proteins found upregulated in acid exosomes with the relative gene expression in cancer tissue samples versus Overall Survival (OS) rates of patients with metastatic melanoma (Table 1).

### Immunohistochemical staining

Tissue sections from primary cutaneous and metastatic lymph node melanoma samples embedded in paraffin were dewaxed and rehydrated. For immunolocalization studies slides were first subjected to heat-mediated antigenic retrieval (10 mM Sodium Citrate buffer pH 6.0) and then to melanin bleaching (warm 10% H2O2). Subsequently slides were permeabilized (0.1% Triton X-100 for 10 min) and saturated (3% BSA for at least 2 h) at RT. After incubation with primary antibody O/N at 4 °C (anti GSN ab75832, 1:100, anti CFL AP08086PU-S Origene and anti HYOU1 ORP150/HSP12A NBP1–32140 Novus 1:50) in humidified chamber, slides were incubated with specific fluorophore conjugated secondary antibodies (Alexa Fluor, Molecular Probes Eugene, OR, USA) for 45 min at RT. Ki67 (M7240 Clone MIB-1, Dako) was used as positive immunostaining control. Negative controls were performed by omission of the primary antibody in each experiment. Finally, slides were mounted with SlowFade anti-fade reagent containing DAPI (Molecular Probes, Eugene, OR, USA) and analyzed by Olympus F1000 laser-scanning confocal microscopy (Olympus,Tokyo, Japan).

### Statistical analysis

Differences were statistically evaluated using Student’s t test. *p* < 0.05 was regarded as significant. In some experiments one-way ANOVA multiple comparison analysis with Tukey’s post test was used. For OS analysis, Mentel-Cox *p*-values < 00.5 were considered as statistically significant.

## Results

### Effect of acidic pH on melanoma morphology

It is known that melanoma is able to grow in a context of extracellular acidosis within the range of pH 6.4 to 7.3 [1, 2]. Trying to closely mimic in vitro the action of tumor microenvironment, we cultured melanoma cells in acidified media at pH 6.7 and pH 6.0, and analyzed the influence of these low pHs on differently staged melanomas, specifically the early primary (EP) and the metastatic non invasive (MNI) cell lines.

**Fig. 1:**
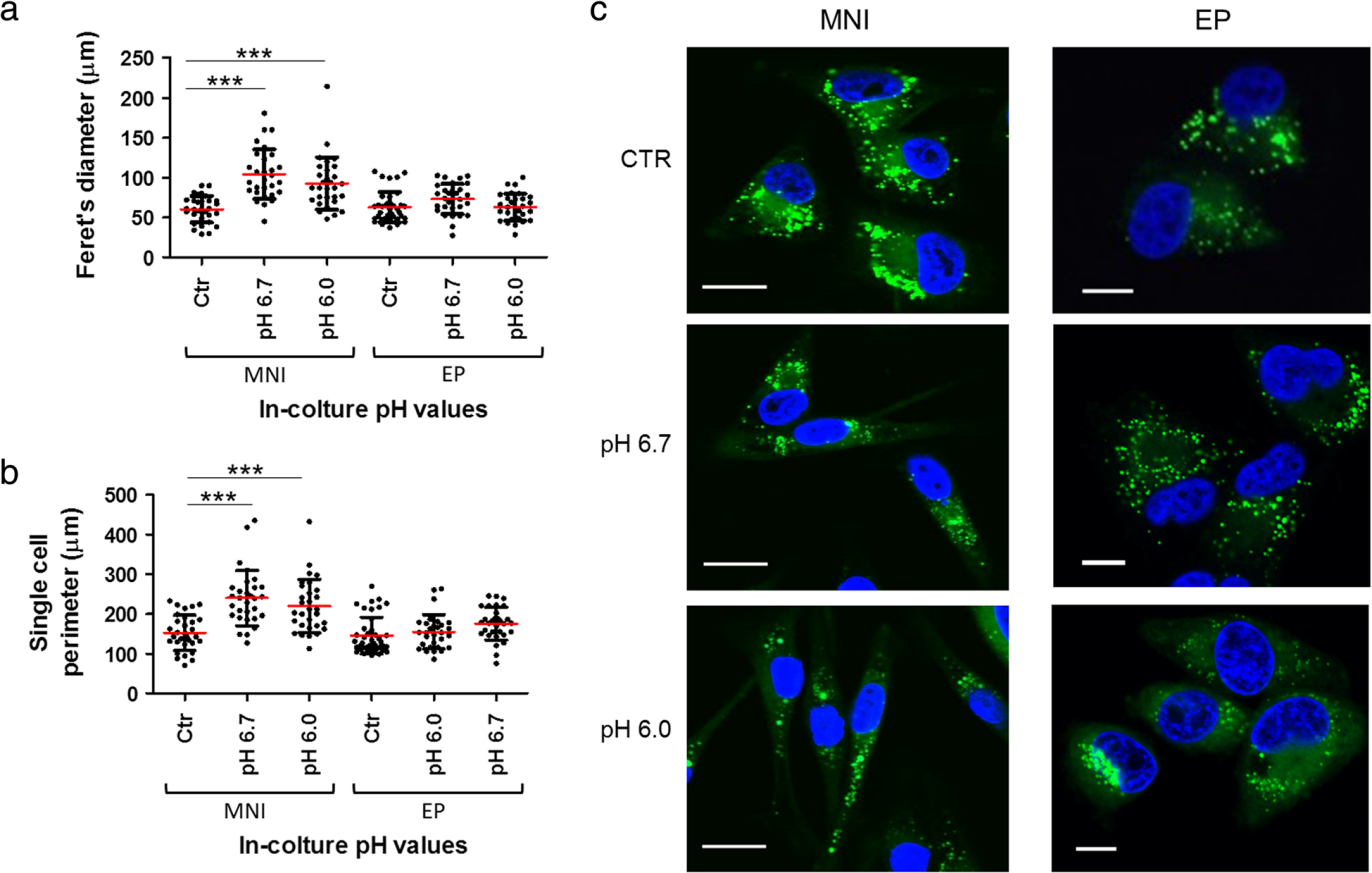
Effect of pH treatments on MNI and EP morphology and C_16_ intracellular distribution. MNI and EP cells were cultured with standard (ctr), pH 6.7 or pH 6.0 culture media for 24 h. Analysis of cell elongation (**a**) and perimeter (**b**) was performed by the determination of Feret’s diameter and cell perimeter of each cell displayed in Additional file 4. **c** MNI and EP cells were labeled with C_16_ for 5 h and incubated at the indicated pHs for 24 h. Cells were afterwards fixed and analyzed by fluorescence under confocal microscopy. The nucleus was visualized with DAPI staining (blue). Scale bars, 10 μm. ***: *p* < 0.0001

In line with previous study [4], microenvironmental acidity did not modify cell viability, whereas it induced in MNI cells a spindle shape morphology after 24 h incubation, being cells significantly more elongated at pH 6.0 and pH 6.7, when compared to controls grown at standard conditions (Fig. 1 a, b, and Additional file 4). Interestingly the EP melanoma morphology was not affected by acidic pHs, thus likely indicating this early stage to be less sensitive to pH variations.

Then we labeled MNI and EP melanomas with green fluorescent hexadecanoic acid (Bodipy FL -C_16_), and analyzed the intracellular distribution, before the isolation of fluorescent exosomes from culture media. After 24 h incubation either at pH 7.4 (ctr) or acidic pHs (pH 6.7 or pH 6.0), cell fluorescence appeared either diffuse in the endoplasmic reticulum area or restricted in spots (Fig. 1c), likely resembling ER/late endosomal/MVB compartments [18]. Interestingly such fluorescence was absent from plasma membrane and was not affected by medium pH.

In line with the morphometric analysis, only MNI cells appeared gradually elongated, i.e. responsive to acidic pHs. Thus, we performed the following experiments on MNI cells.

Next, to exclude the presence of lipid probe aggregates, we checked the effective C_16_ incorporation and transformation in cell major lipid classes and the effects, if any, resulting from pH treatments. TLC analysis showed the same amount of major membrane lipid classes independently from pH treatment (Additional file 5).

### Biophysical and biochemical properties of C_16_-exo are not affected by acidic pH

**Fig. 2:**
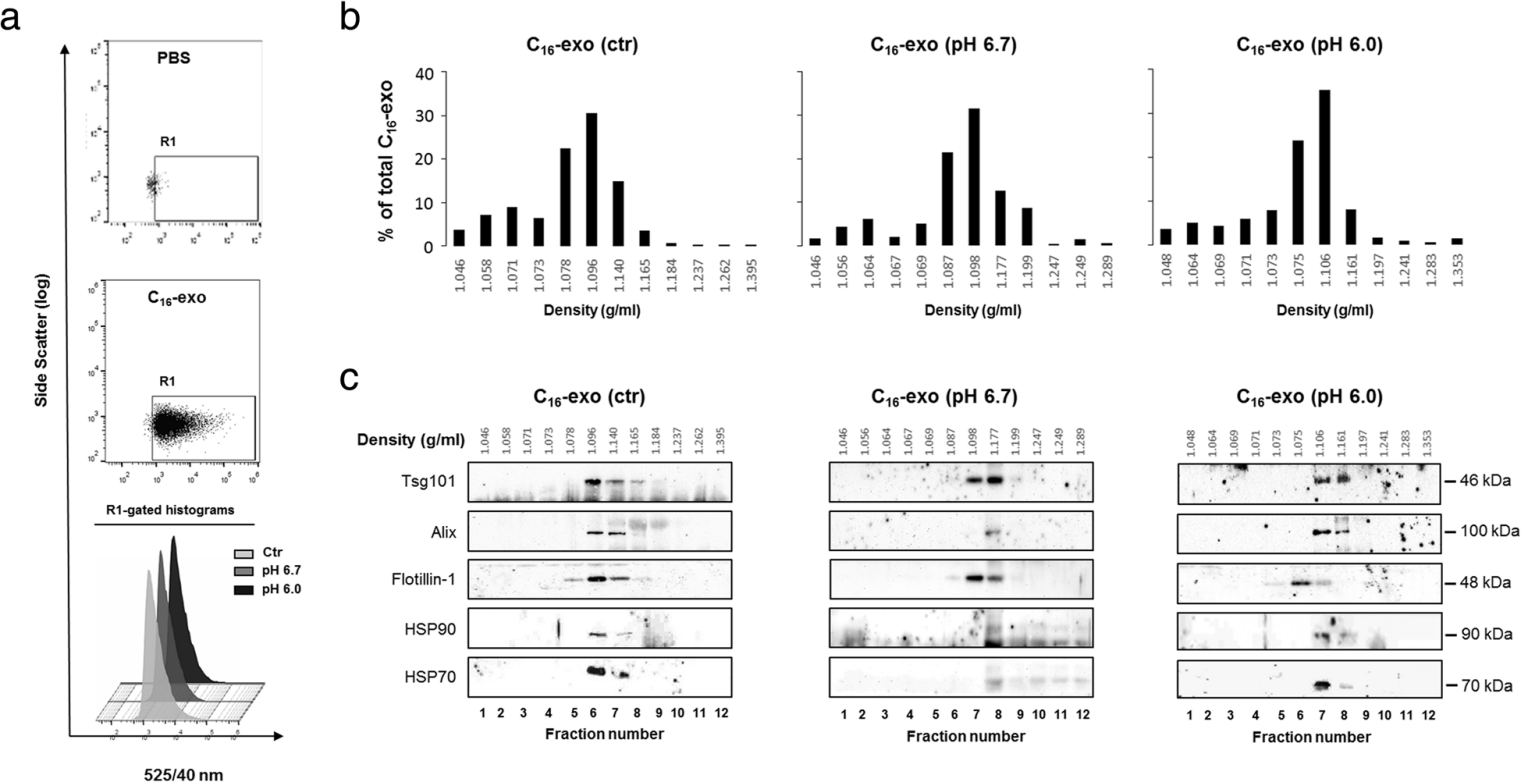
FACS analysis of C_16_-exo and distribution on iodixanol gradient. **a** FACS analysis of C_16_-exo deriving from MNI cells cultivated with medium at different pHs To design the R1 region above instrument background noise only PBS was acquired (upper panel). Note that no events were acquired in this region. (mid panel) example of exosome population. (Lower panel) Histograms representing the green fluorescence intensity distribution of events gated in R1 regions of exosome samples recovered in control, pH 6.7 and pH 6.0 conditions **b** C_16_-exo (ctr) (2,5 × 10^8^), C_16_-exo (pH 6.7) (1,8 × 10^8^), C_16_-exo (pH 6.0) (2,5 × 10^8^), were loaded at the bottom of iodixanol gradient and subjected to ultracentrifugation for 19 h. For each condition, 2 μl of resulting fractions were FACS counted and each fraction represented as percent of total C_16_-exo recovered. Fraction densities were determined by refractometry. **c** An equal volume of each fraction was analyzed for the indicated exosome markers by western blotting. Data shown are representative of three independent experiments

Fluorescent exosomes (C_16_-exo) were recovered from MNI conditioned medium after 24 h cell culture according to an ultracentrifugation-based protocol (Additional file 2). The resulting pellet was directly analyzed by FACS, and showed the presence of a single fluorescent population indicated as C_16_-exo without any background noise on FL1 channel, as depicted by R1 region of the PBS condition (Fig. 2a). pH treatments did not alter biophysical vesicles parameters, since C_16_-exo displayed homogeneous fluorescence intensity (Fig. 2a, histograms).

A parallel TLC analysis of C_16_-exo confirmed the presence of fluorescent major membrane lipid classes, being similar in composition, with slight differences in fluorescence intensities at low pHs (Additional file 5).

To gain insight into the quality of vesicles secreted at acidic pHs we used a protein specific dye, Carboxyfluoresceindiacetate succinimidyl ester (CFDA-SE), generally used as marker of integrity of protein loaded vesicles [35].

To ensure that the vesicle esterase activity was not affected by pH, we tested the cell esterase function after 24 h of pH treatments. Cell fluorescence was not modified by acidic pHs (Additional file 6). Vesicles pellet obtained from MNI cells was incubated with CFDA-SE to obtain CFSE-EV, as depicted in Additional file 6. FACS analysis indicated the presence of a single population as for C_16_ labelling, and fluorescence intensity was not affected by pH treatments (Additional file 6).

This result confirmed that vesicles secretion at acidic pHs was referred to intact protein-loaded structures. To confirm the identity of C_16_-exo population as exosomes, we evaluated the co-occurrence of three main parameters: fluorescent vesicles, density and expression of specific markers after gradient flotation. To avoid the risk of artifacts deriving from osmotic damages to the vesicles, we opted for an iodixanol gradient.

C_16_-exo isolated from untreated (ctr) or acid (pH 6.7, pH 6.0)-treated MNI cells were subjected to 10–40% density gradients. Results indicated in all conditions the existence of a population mainly distributed in fractions corresponding to flotation densities between 1.096 and 1.177 g/ml specific to exosomes [14] (Fig. 2b), and positive for the well known exosome markers Tsg101, Alix, HSP90, flotillin-1, and HSP70, [16, 36] (Fig. 2c).

In each condition, approximately 15% of C_16_-vesicles floated at lighter densities (1.046–1.073 g/ml), and was devoid of exosome markers, possibly indicating the existence of a lighter endosomal vesicle population with a lower protein content.

### Enhanced C_16_-exo secretion at acidic pH depends on melanoma stage

The amount of secreted exosomes on the base of disease stage in melanoma is still a question of debate. To address this point, we used the above-described strategy by comparing C_16_-exo secretion from four differently staged melanoma cell lines, early primary (EP), metastatic non-invasive (MNI), primary/invasive (PI) and metastatic invasive (MI). To better define the stage of the melanoma cell lines used, we summarized the classification and gene mutations, and evaluated whether molecules related to melanoma progression, such as E-, and N-cadherin involved in epithelial-mesenchymal transition (EMT), Tyrosinase and AP2α were differently expressed (Additional file 1 and Additional file 14). Results indicated the occurrence of EMT together with low expressions of AP2α and Tyrosinase in PI and MI cells, in line with an advanced tumor stage. On the contrary EP and MNI being in a pre-EMT phase, showed a higher expression of AP2α and Tyrosinase, thus indicating an earlier stage in tumor progression. Next, we evaluated to what extent and at which disease stage acidic pH could significantly influence exosome secretion.

C_16_-exo were recovered from melanoma cell lines cultured at pHs 6.0 and 6.7 for 24 h and FACS counted. The acidity of the medium significantly enhanced exosome secretion when compared to buffered medium (ctr) in MNI cells.

**Fig. 3:**
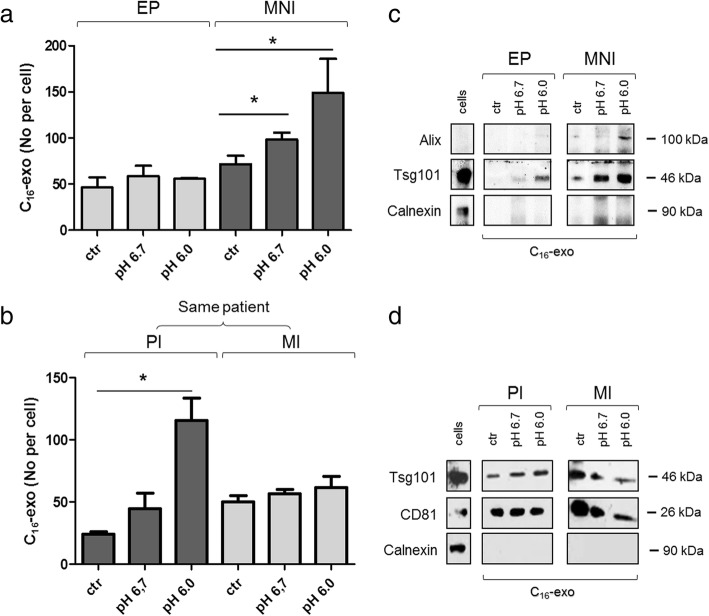
Analysis of C_16_-exo secreted in control or acidic conditions. **a** and **b** EP, MNI, PI and MI cells were treated with C_16_ (7 μm) for 5 h and then cultured in control or acid (pH 6.0 or pH 6.7) medium for 24 h. Hereafter conditioned medium was subjected to ultracentrifugation for exosome isolation. Fluorescent vesicles were then counted by FACS. The graph shows the number of secreted C_16_-exo per cell. **c** and **d** Western blot analysis of exosome for the presence of principal exosome markers (Alix, Tsg101 and CD81) and the absence of Calnexin, a marker of ER used as a negative control. Data shown are representative of three independent experiments. * *p* < 0.05

On the contrary, acid treatment did not affect exosome release in EP melanoma (Fig. 3a). One explanation might be that the acid treatment does not influence exosome secretion in early primary melanoma due to its intrinsic scarcely aggressive nature.

Same analyses conducted on PI and MI cell lines, obtained from the same patient, confirmed the higher exosome secretion in advanced versus primary melanomas. Specifically, in standard culture condition MI produced 50 exosome per cell versus 25 secreted by PI, in line with its more aggressive nature (Fig. 3b). However in this case acidic pH positively influenced exosome release in primary, but not in metastatic cell line. This suggests that acidic pH can affect exosome secretion at a specific tumor developmental stage, i.e. only when the massive secretion of specific molecules is required for the spread of the tumor and the progression of the disease.

Accordingly, the pH-sensitive PI cell line is already endowed with invasive capability, thus indicating a more advanced stage with respect to EP not responsive to acidic pH in terms of exosome release. The identity of these vesicles as exosomes was confirmed by the expression of the common exosome markers Tsg101, Alix and CD81 and the absence of calnexin (Fig. 3c, d) [37].

To investigate a potential trigger to enhance exosome secretion we first evaluated intracellular exosomal markers expression (Alix and Tsg101) in the acid pH-responsive (MNI) and not responsive (MI) cell lines. We found that Alix was significantly upregulated after short time pH 6.0 exposure and decreased together with Tsg101 after 24 h in MNI cells (Additional file 7), thus suggesting the enhanced exosome release as the result of an augmented biosynthesis. Interestingly, these markers were found unchanged during 24 h acid treatment in MI cells (Additional file 7), in line with their unresponsiveness to acid pH.

We further evaluated intracellular pH variations as responsible of augmented exosome release. In fact, it is known that external pH acidification can induce variations in intracellular pH [38], favouring cell proliferation, drug resistance and metastasis progression [5, 39]. Thus, we checked the intracellular pH after 24 h acid pH treatment in MNI and MI cells (Additional file 7). In basal condition both cell lines displayed a pHi ranging between 6.8–6.9, that was slightly increased at pH 7.0 by extracellular acidification only in MI cells. However, the pHi of MNI cells was not affected by extracellular acid pH, indicating that increased exosome secretion does not correlate with intracellular pH variations.

Altogether, these findings suggest that melanoma stage is an important requisite for acidic pH-responsiveness, that is mediated by an increase in exosome biosynthesis and release.

### Acid C_16_-exo induces migration and invasion

Increase in cell migration and invasion is hallmark of tumor advancement [40]. To evaluate whether acid exosomes could play a role in melanoma progression, we performed a set of experiments on pH-sensitive metastatic not invasive melanoma.

**Fig. 4:**
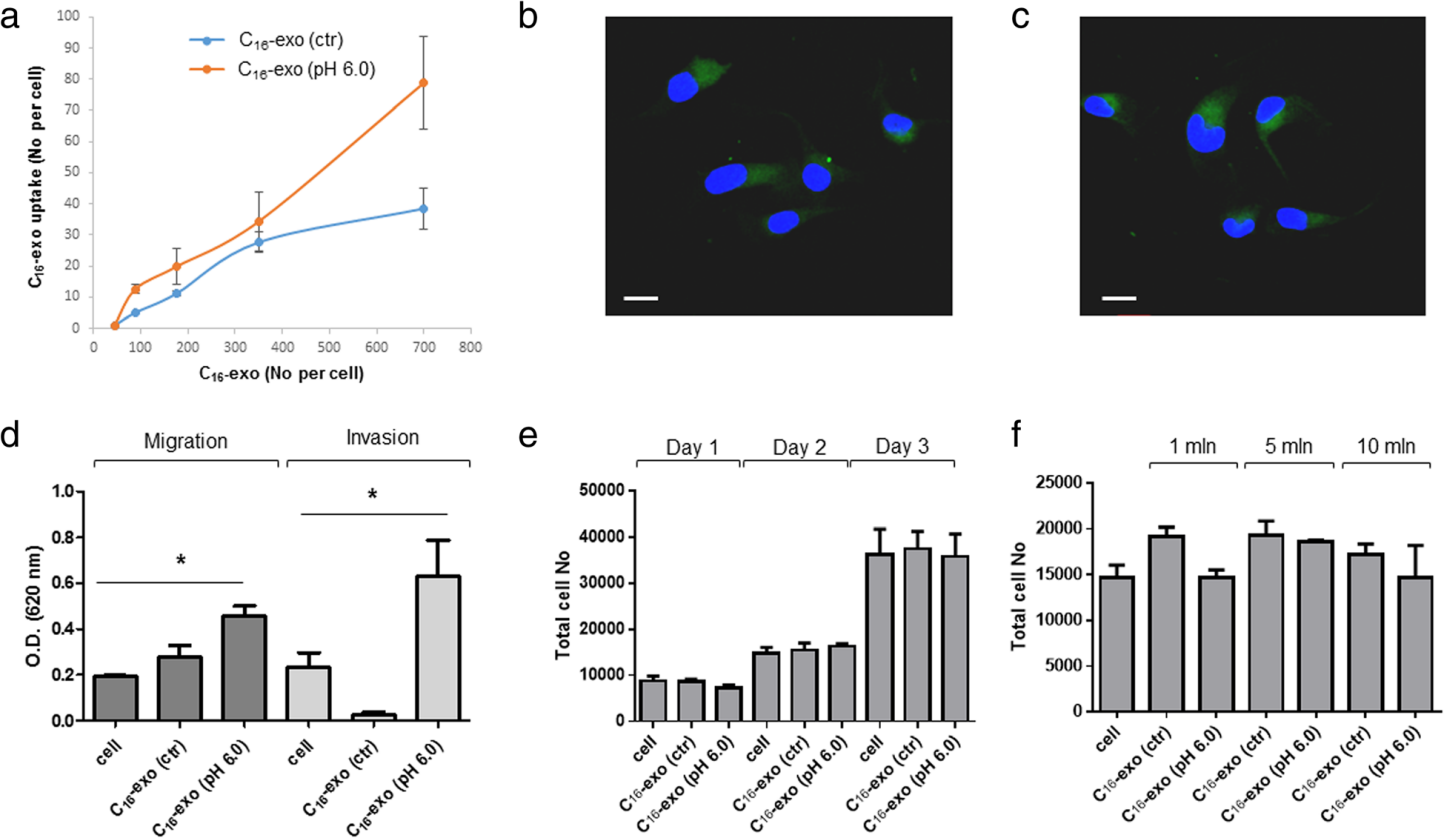
C_16_-exo uptake and functional assays. **a** MNI cells were labeled with C_16_ and incubated for 24 h with ctr or pH 6.0 medium. C_16_-exo (ctr) and (pH 6.0) were recovered and incubated at increasing doses for 2 h at 37 °C with MNI cells at pH 7.4. Cell fluorescence was analyzed by FACS. The number of incorporated exosomes per cell was calculated and represented in the graph. Points: mean ± S.D. (*n* = 4). **b** and **c** Confocal microscopy analysis of MNI cells after 2 h incubation with C_16_-exo (2 × 10^3^ per cell) (ctr) (**b**) or (pH 6.0) (**c**). DAPI stains show the nucleus (blue). Green: C_16_-exo. Scale bars, 10 μm. **d** Migration and invasion assays of MNI cells (3.5 × 10^4^) after incubation with (10 × 10^6^) C_16_-exo, deriving from (ctr) or (pH 6.0) treated MNI cells. Migration and invasion were evaluated after 72 h of incubation by a colorimetric assay at 620 nm. * *p* < 0.05. **e** and **f** Cell viability was assayed by trypan blue exclusion of dead cells. MNI cells (2 × 10^3^) were incubated with C_16_-exo (ctr), (pH 6.7), (pH 6.0) (5 × 10^6^) and counted at the indicated days, or with 1 × 10^6^, 5 × 10^6^, 10 × 10^6^ C_16_-exo (ctr), (pH 6.7), (pH 6.0) and counted after two days. Histograms: means ± S.D. (*n* = 3). Results indicate that in **e** and **f** the differences among ctr and pH 6.0 are not statistically significant

Thus we first determined the rate of control and acid exosome uptake measured as number of vesicles transferred in target cells at pH 7.4. Results indicated a slightly higher transfer efficiency of acid C_16_-exo (pH 6.0), that was constantly reproduced at increasing vesicle doses (Fig. 4a). Both classes of captured exosomes were visible in cytoplasmic area around nucleus (Fig. 4b-c), and their transfer was influenced by extracellular pH. In fact, both classes of exosomes were more efficiently transferred at extracellular acid pHs (Additional file 8), with the exception of acid exosomes on cells at pH 6.0. In particular, the acid exosome uptake at pH 6.0 was significantly lower of control exosomes uptake at pH 7.4, thus further accounting for the greater extracellular availability of acid exosomes above reported.

Interestingly, in EP cells the rate of control and acid exosomes was similar and the amount of acid exosomes transfer was not influenced by extracellular pH (Additional file 8),

Next, the functional role of control and acid exosomes from MNI cells was explored through in vitro migration and invasion assays. MNI melanoma cells were incubated for 72 h with the same amount of exosomes (1 × 10^7^) released from control or pH 6.0-cultured cells. The significant increase in cell migration and invasion observed after uptake of acid C_16_-exo (Fig. 4d), raised the hypothesis that a specific molecular content could be responsible of cell acquired aggressiveness.

To exclude the enhanced cell migration and invasion as the result of increased proliferation, we examined the effects of C_16_-exo (ctr) and C_16_-exo (pH 6.0) on MNI cell growth. After 72 h incubation, cell proliferation was not affected upon exosome treatments either in time and dose-response experiments (Fig. 4e, f).

To address whether these results could mirror the in vivo process where a slow acclimation at low pH is required to select resistant cells [4], we subjected MNI cells to a two months low pH culture, followed by 1 month re-acclimation at physiological pH (Additional file 9). In line with 24 h pH 6.0 treatment, we observed an elongated cell morphology, and acquired migratory ability in acid selected condition. Exosomes obtained from acid selected cells induced migration in control cells, thus confirming a stable pro-migratory role exerted by acid microenvironment through exosomes.

### Acid exosomes display an enrichment in metabolic pathways related to tumor aggressiveness

Looking for the different molecular cargos potentially responsible of the newly acquired metastatic properties, we investigated the protein composition of exosomes secreted by MNI cells under acidic pressure through a label-free quantitative proteomics.

Exosomes recovered after 24 h of cell culture at acidic (pH 6.0) and buffered (ctr) conditions were run on 4–12% SDS-PAGE and subjected to in-gel trypsinization. The extracted peptides were then analyzed by Nano-RPLC. A total number of 186 and 157 proteins were identified in pH 6.0 and control samples, respectively.

The presence of 24 proteins (pH 6.0) and 21 proteins (ctr) matching some of the most frequently identified proteins within exosomes [41] further validated our vesicle preparation (Additional file 10). Moreover, the lack in our lists of melanosome markers, such as DCT and GPNMB, excluded any possible contamination with melanosomes, known to be abundantly secreted from melanoma cells [42].

**Fig. 5:**
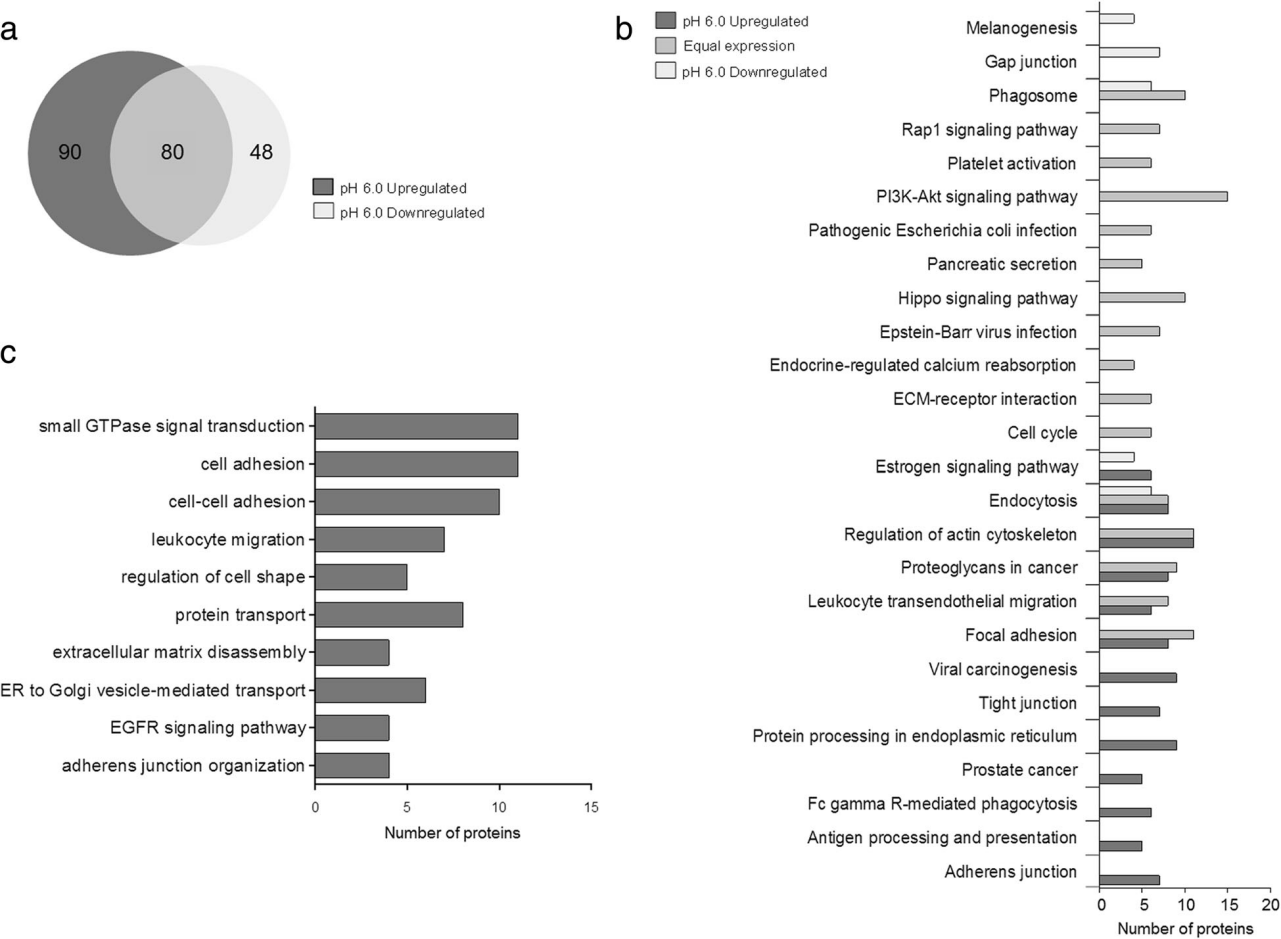
Proteome characterization by HPLC-MS/MS of control and pH 6.0 exosome obtained after ultracentrifugation. **a** Venn diagram depicting the overlap of upregulated and downregulated pH 6.0 proteins with respect to control condition. **b** Functional analysis of the downregulated, equally expressed and upregulated proteins at pH 6.0. Only over-represented metabolic pathways with *P* ≤ 10^− 2^ were considered. **c** GO enrichments of pH 6.0 upregulated proteins. Only over-represented categories with P ≤ 10^− 2^ were considered

To gain insights into specific protein signature at acidic pH, we generated three list of up-, equally and down-regulated proteins in the pH 6.0 condition according to the ratio of normalized emPAI values. We found that 24 h cells incubation at low pH was sufficient to modify exosome protein profile, being more than 50% of the proteins up-regulated, as displayed by the comparative Venn diagram (Fig. 5a).

Functional annotation analysis of these three data sets was performed interrogating KEGG pathway database (Fig. 5b and Additional file 11). Interestingly, in acid conditions functional categories, such as proteoglycans, focal adhesion and protein processing in endoplasmic reticulum, responsible for melanoma migration and invasion, metastasis and survival [21–23], resulted over-represented.

Moreover, proteins related to melanogenesis were found down-regulated in acid exosomes, thus confirming a cell acquired more aggressive properties in line with a reduced melanin content.

Further analyses by using Gene Ontology [43] confirmed the enrichment of acidic pH up-regulated proteins in some key biological processes (Fig. 5c and Additional file 12) as cell-cell adhesion, leukocyte migration, regulation of cell shape, small GTPase mediated signal transduction, EGFR signaling pathways, all associated with the migratory properties of the cells.

Taken together, these results indicate that exosomes released at low pH contain specific proteins that upon transfer to recipient cells can mediate melanoma malignancy.

### Acid exosomes molecules profile reflects gene expression in metastatic melanoma patients

To find a clinical relevance of modified exosome content at acidic pH, we performed a meta-analysis of all the metastasis-related functional categories listed in Additional file 12, by using Prognoscan database [24] (Table 1).

**Table 1:** Meta-analysis of upregulated protein in acid exosomes through PrognoScan

Gene	GenBank ID*	Probe name	Mentel-Cox *p*-value**
HRAS	3265	212983_at	0.010599
IQGAP1	8826	213446_s_at	NS
ACTN1	87	200601_at	NS
ACTN4	81	208636_at	NS
CDC42	998	214230_at	NS
CFL1	1072	1555730_a_at	NS
CFL2	1073	233496_s_at	0.002532
FN1	2335	210495_x_at	NS
NRAS	4893	202647_s_at	0.002301
GSN	2934	202647_s_at	0.044629
VCL	7414	200930_s_at	NS
CD44	960	229221_at	NS
TIMP3	7078	201147_s_at	0.029350
THBS1	7057	201110_s_at	NS
TLN1	7094	203254_s_at	NS
CTNND1	1500	1557944_s_at	NS
ICAM1	3383	215485_s_at	NS
DNAJA2	10,294	209157_at	NS
GANAB	23,193	214626_s_at	0.003695
HSP90AA1	3320	211968_s_at	NS
HSP90B1	7184	216449_x_at	0.040481
HSP90AB1	3326	200064_at	0.029072
HSPA1L	3305	233694_at	0.048446
HSPA5	3309	230031_at	0.026684
HYOU1	10,525	200825_s_at	0.037596
VCP	7415	214990_at	NS

*publicly accessible at https://www.ncbi.nlm.nih.gov/gene

**Numbers depict Mentel-Cox *p* values for gene expression with significant difference in patient’s overall survival. Only values with p < 0.05 are indicated. NS, patient’s overall survival not significant (p > =0.05) for the indicated high or low gene expression. The analysis was performed by interrogating PrognoScan database for gene expression in cancer tissue samples versus overall survival rates of patients with metastatic melanoma. All the listed genes refer to proteins involved in metastatic processes found upregulated in acid exosomes (Additional file 12). The analysis has been performed by using the dataset GSE19234, publicly accessible at GEO database [34]

**Fig. 6:**
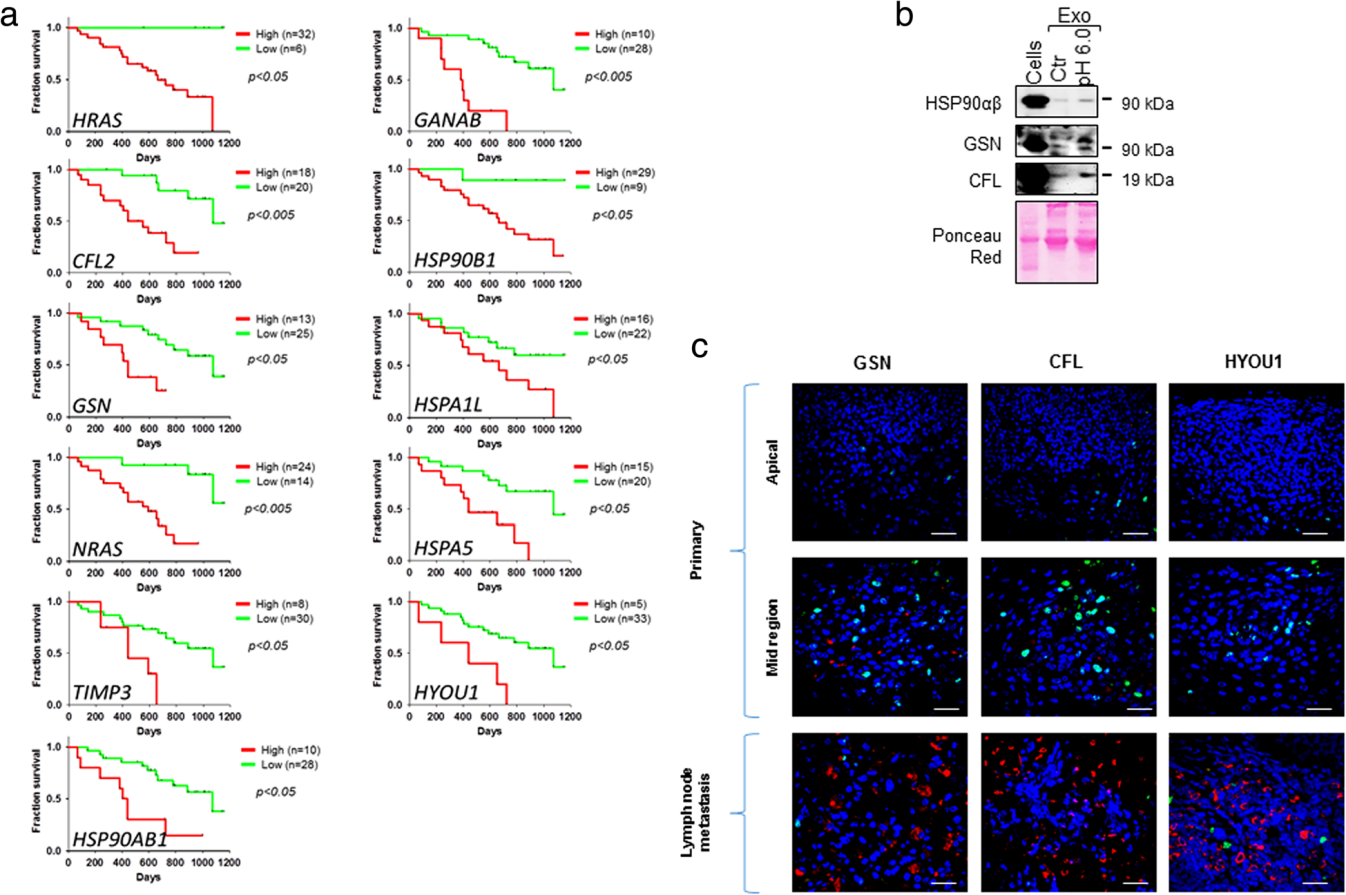
Meta-analysis of significant metastatic upregulated proteins in acid exosomes through Prognoscan. **a** Kaplan-Meyer curves depicting the Overall Survival of metastatic melanoma patients with high (red lines) or low (green lines) expression of the genes listed in Table 1 that resulted statistically significant. Mentel-Cox *p*-values for each plot are showed. **b** Western blotting analysis of ctr and pH 6.0 exosomes (20 μg). Cell lysate was used as positive control. Expression of CFL, GSN and HSP90αβ is shown. Ponceau red is shown for equal exosome protein amount. **c** confocal immunofluorescence analysis with the indicated antibodies on primary (apical and mid region), and metastatic melanoma biopsies from the same patient. Ki67 labeling was used as internal control. Red, CFL, GSN, HYOU1; blue, DAPI; green, Ki67. Bar 50 μm

Interestingly around 50% of these genes, represented by HRAS, GANAB, CFL2, HSP90B1, HSP90AB1, GSN, HSPA1L, NRAS, HSPA5, TIMP3, HYOU1, showed a significant Mentel-Cox-*p*-value, i.e. a positive correlation of their high expression in cancer tissues and poor prognosis in patients with metastatic melanoma (Fig. 6a). A subset of these genes was checked for gene expression levels in MNI cells, and was found upregulated after pH 6.0 treatment (Additional file 13).

This data was further confirmed by the expression of a representative set of these genes, i.e. HSP90αβ, CFL, GSN in exosomes released at acidic pH. Same amount of control or acid exosomes was analyzed by western blotting. As expected, the upregulation of all proteins tested was evident in acid exosomes compared to control (Fig. 6b).

Finally, the expression levels of CFL, GSN and HYOU1 proteins were evaluated on some representative bioptic samples from melanoma patients. Interestingly, a general increment of these proteins was observed in lymph node autologous metastases compared with their primary counterparts (Fig. 6c).

All together, these data indicate that molecular cargo of exosomes released under microenvironmental acidity mirrors the protein content of metastatic lesions.

This may suggest acid exosomes as a diagnostic tool of poor prognosis in melanoma patients.

## Discussion

To study molecular mechanisms involved in the early steps of melanoma progression, we analyzed four differently staged human melanoma cell lines, reflecting melanoma progression, in the context of their most frequently occurring microenvironmental change, i.e. pH acidification. Microenvironmental acidic pH exerts multiple roles in tumor advancement that imply local invasion and dissemination of cancer cell [44, 45], activation of MMPs [46], and impairment of the immune response [47].

In the melanoma model, external low pH was found to promote metastasis [5] and to affect exosome traffic [7]. An exosome functional role was principally described in the later stages of melanoma progression, being exosomes involved in the production of long-distance metastases [12]. Nonetheless, a role of exosomes in driving metastatic properties at intratumoral level under acidic pressure was never defined so far.

A study on the effect of acidic pH variations has already been performed, but it was limited to the description of the increase of exosome protein content [7]. In this paper thanks to a new cell labeling methodology [18], we could monitor exosome amount through quantification of intact particles under microenvironment pH fluctuations, naturally occurring in metastatization process.

To more closely reproduce in vitro such pH fluctuations, we set up two experimental conditions, i.e. pH 6.7 and pH 6.0. At these conditions, we monitored exosome release among the plethora of extracellular vesicles secreted from melanoma cells [48], by using a technique based on the generation of nascent fluorescent vesicles of endosomal origin, considered *bona fide* exosomes (C_16_-exo) [18].

We definitely assessed that in MNI cell line culture at acidic pH was recovered an increased number of vesicles compared to that secreted at pH 7.4. This was not correlated with intracellular pH variations, but was due to an elevated exosome biosynthesis and reduced re-uptake.

This new labeling technique offered us an eligible and innovative method for melanoma exosome detection and analysis. In fact, we could estimate that the enhanced C_16_-exo secretion upon pH treatment was effective, and referable to small and intact structures.

In general, the increased amount of secreted exosomes represents a hallmark of disease stage advancement. However, in melanoma this issue was not completely clarified, being reported in some studies an increased amount of exosomes in plasma from advanced patients [49, 50], and in other studies similar numbers of exosomes in patients at different clinical stages [12, 51].

To address this issue we monitored C_16_-exo secretion from a panel of primary and metastatic melanomas. We found: 1) a higher exosome number released by metastatic than primary melanomas; 2) acidic pH increases exosome release in melanoma at an intermediate stage (i.e. not early primary or metastatic),

It is conceivable that increased extracellular availability of exosomes at this stage is crucial for the progression of the disease at a step in which the maximal spread of newly acquired and specific molecular information are needed to drive and sustain tumor aggressiveness. To confirm such hypothesis, we tested the tumor promoting role of acid released C_16_-exo on MNI cells. We found that C_16_-exo released by MNI melanoma kept at low pH exerted a pro-migratory and invasive role on autologous pH *naïve* cells.

Interestingly, although control and acid exosomes are greatly taken up by melanoma cells at extracellular acid pHs, only those secreted at low pH are able to induce into the less aggressive cells distinctive migratory and invasive skills. This property can be maintained also after long-term acid pH selection and re-acclimation at pH 7.4, in line with the in vivo continuous acid exposure.

Accordingly, a comparative proteomic analysis of exosomes released at pH 6.0 versus control, indicated in acidic exosomes a general increment in the expression of some protein categories as those belonging to focal adhesion, actin cytoskeleton regulation, leukocyte trans-endothelial migration, or more specifically to those proteins governing the modification of cell morphology such as small GTPase mediated signal transduction, and regulating pro-migratory pathways such as EGFR.

Most of these molecules were already described in metastatic exosomes [51], and some of them (HRAS, GANAB, CFL2, HSP90B1, HSP90AB1, GSN, HSPA1L, NRAS, HSPA5, TIMP3, HYOU1) were found upregulated in patients with poor prognosis. Among this set of genes, we here validated the higher expression of CFL, GSN and HYOU1 in metastatic site with respect to autologous primary tumor (patient with Clark’s level IV), whereas Hsp90 protein overexpression was previously described [52]. Altogether, these results highlight the prognostic value of acid exosomes molecular cargo.

Due to the different nature of these molecules, i.e. proto-oncogenes (HRAS, NRAS), metalloprotease (TIMP3), heath shock protein isoforms (HSP90AB1, HSP90B1, HSPAIL, HSPA5), enzyme (GANAB) involved in protein folding and control in endoplasmic reticulum, and actin-binding proteins (GSN, CFL2) regulating reorganization of actin filaments, we can speculate independent functional roles occurring in cell transformation, and ultimately leading to enhanced cell aggressiveness.

However, we can hypothesize a prominent role for GSN and CFL in inducing cell migration. In fact, it is feasible that both proteins, imported into recipient cells, elicit actin cytoskeleton reorganization, thereby directly modulating the migratory behavior of melanoma cells.

In melanoma progression a role of tumor surrounding normal cells, such as stromal or endothelial cells, can not be excluded. In fact, exosomes released by cancer cells are able to induce angiogenesis by transferring their content to endothelial cells [53–55], and also trigger fibroblast differentiation into myofibroblast [56].

Moreover, exosomes derived from metastatic melanoma have been shown to facilitate the formation of pre-metastatic niche by educating bone marrow derived cells [12].

Thus, it is likely surmised that acid exosomes can play a role also in remodeling a tumor microenvironment through the conditioning of stromal cells, that may induce microenvironmental adaptation and placement for metastatic melanoma to take place. However, this study will be the object of future work.

We here described a stage-specific melanoma pro-invasive feature acquired from acid pH exposure, and exerted through exosomes enriched in HRAS, GANAB, CFL2, HSP90B1, HSP90AB1, GSN, HSPA1L, NRAS, HSPA5, TIMP3, HYOU1 genes, that were statistically related to melanoma patients poor prognosis.

The model system here proposed could be hypothesized in vivo, i.e. within the heterogenic tumor mass, where only some cells might initiate to highly produce and disseminate exosomes in response to acidification, so far influencing the behavior of neighboring cells.

## Conclusions

Our findings contributes to a better understanding of the role of exosomes in a specific stage of melanoma progression driven by extracellular acidity. Overall, the specific content of exosomes produced under acid conditions may represent a melanoma stage-specific signature, which could be the object of new target therapies against melanoma development.

## Additional files

Additional file 1: **Figure S1.** Characterization of cell lines used in this study. a Overview table depicting classification and gene mutations. b Western blotting analysis of total cell lysates (20 μg/lane), with the indicated antibodies. (PNG 369 kb)

Additional file 2: **Figure S2.** Experimental workflow of C_16_-exo isolation from MNI cell culture. (PNG 271 kb)

Additional file 3: Additional Methods. (DOCX 22 kb)

Additional file 4: **Figure S3.** a MNI, and b EP cells phase contrast with bounds of the selected cells (*n* = 30) for each condition. The lower microphotographs depict the selected cells by means of the Region of Interest (ROI, yellow lines) plugin of ImageJ. The selected cells were analyzed for elongation and perimeter by the determination of Feret’s diameter. (PNG 1662 kb)

Additional file 5: **Figure S4.** TLC analysis of fluorescent lipids of MNI cells and exosomes. a Cells at the indicated pHs (above) and quantification values (below) of each depicted peak (arrows) as percentage value within each lane. b Exosomes at the indicated pHs (above) and quantification values (below) of each depicted peak (arrows) as percentage value within each lane. Sphingomyelin (SM), cardiolipin (CL), phosphatidylserine (PS), phosphatidylinositol (PI), phosphatidylethanolamine (PE), phosphatidylcholine (PC) and bis(monoacylglycero)phospahate (BMP). (PNG 410 kb)

Additional file 6: **Figure S5.** MNI cells labeling with CFDA-SE, vesicles isolation and FACS analysis. a MNI cells were left untreated, or pH 6.7, and pH 6.0 treated for 22 h followed by 0.3 μM CFDA-SE labeling 5 min at room temperature, then FACS analyzed. Bars: mean ± S.D. (*n* = 3). b Workflow of vesicles isolation from MNI cells. Vesicles were labelled with (10 μM) CFDA-SE for 1 h at room temperature to obtain CFSE-EV. **c** FACS analysis of CFSE-EV population deriving from MNI cells cultivated at different pHs. To design the R1 region above instrument background noise only PBS was acquired. Note that no events were acquired in this region. Histograms represent the green fluorescence intensity distribution of events gated in R1 regions of CFSE population recovered from MNI cells in standard culture condition (ctr), pH 6.7 and pH 6.0. (PNG 383 kb)

Additional file 7: **Figure S6.** MNI and MI intracellular expression of exosome markers and intracellular pH (pHi) evaluation a-c. MNI, and d-f MI cells were left untreated or pH 6.0 treated for the indicated times, then cells were lysed and 30 μg/lane analyzed for Alix and Tsg101 by western blot. a, d representative western blots are showed. b, e Alix, and c, f Tsg101 expression were normalized against tubulin by densitometry analysis and expressed as fold increase (n = 3),. g pHi measurement. Cells were left untreated or incubated at pH 6.0. After 24 h intracellular pH was measured with 3 μM BCECF-AM for 45 min at 37 °C. Mean ± s.d. (n = 3) *, *p* < 0,05 **, p < 0,01. (PNG 485 kb)

Additional file 8: **Figure S7.** C_16_-exo uptake at extracellular acid pH. 17 × 10^6^ C_16_-exo (ctr) and (pH 6.0) obtained from a MNI cells and c EP cells, were incubated for 2 h at 37 °C with parental cells (0.05 × 10^6^) at the indicated pHs. The number of incorporated exosomes per cell was calculated and represented as percentage of respective control at pH 7.4. b C_16_-exo (ctr) and (pH 6.0) from EP cells were incubated at increasing doses with EP cells at pH 7.4 for 2 h at 37 °C. Cell fluorescence was analyzed by FACS and the number of transferred exosome calculated as described in the text. Points: mean ± S.D. (n = 3). *, p < 0,05; **, p < 0,01; ***, p < 0,005 vs respective pH 7.4 condition; ^§§,^ p < 0,01 vs C_16_-exo ctr at pH 7.4. (PNG 360 kb)

Additional file 9: **Figure S8.** MNI cells selection at acid pH. a Workflow of MNI selection at acid pH. At the end of acclimation cells were assayed for b growth, c morphology (magnification 10×) and d migration. MNI ctr and MNI acid sel. C_16_-exo were obtained as described in the manuscript, and same number (10 × 10^6^) incubated for 72 h with MNI ctr cells. (n = 3) *, *p* < 0.05. (PNG 596 kb)

Additional file 10: **Table S1.** Identification of exosomal markers in melanoma exosomes by mass spectrometry. Table reporting the list of identified exosomal proteins, according to the Exocarta database [39], in control (ctr) or pH 6.0- exosomes from MNI cells. x, proteins identified. (DOCX 17 kb)

Additional file 11: **Table S2.** List of exosomal proteins from MNI cells identified by mass-spectrometry and found upregulated and down-regulated at pH 6.0, or equally expressed in control condition. In case of proteins specific for each condition emPAI_norm_ value is reported. In case of proteins found in both conditions emPAI_norm_ ratio (ρ) is shown. (DOCX 59 kb)

Additional file 12: **Table S3.** pH 6.0 upregulated categories involved in metastatic processes are indicated. Proteins found in these categories are listed. (DOCX 15 kb)

Additional file 13: **Figure S9.** qRT–PCR in MNI ctr and pH 6.0 treated (24 h) cells. Relative gene expression levels were normalized on GAPDH. (PNG 158 kb)

Additional file 14: Additional References. (DOCX 17 kb)

## Abbreviations

CFSE-EV: Extracellular vesicle labelled with CFDA-SE
C_16_: Bodipy FL-C_16_
C_16_-exo: Exosome labelled with Bodipy FL-C_16_
CFL: Cofilin
EMT: Epithelial-mesenchymal transition
EP: Early primary melanoma
ER: Endoplasmic reticulum
EV: Extracellular vesicle
GANAB: Glucosidase II alpha subunit
GSN: Gelsolin
HRAS: HRas proto-oncogene
HSP90AB1: Heat shock protein 90 alpha family class B member
HSPA5: Heat shock protein family A member 5
HSPAIL: Heat shock protein family A member I like
HYOU1: Hypoxia up-regulated protein 1
IHC: Immunohistochemistry
MI: Metastatic invasive melanoma
MNI: Metastatic non -invasive melanoma
NRAS: Neuroblastoma RAS viral oncogene homolog
PI: Primary invasive melanoma
TIMP3: Tissue inhibitor of metalloproteinase 3
