# Supplementary figures and images for: Acidic microenvironment plays a key role in human melanoma progression through a sustained exosome mediated transfer of clinically relevant metastatic molecules
Source: J Exp Clin Cancer Res. 2018 Oct 5;37:245. doi: 10.1186/s13046-018-0915-z (PMC6173926; doi:10.1186/s13046-018-0915-z)

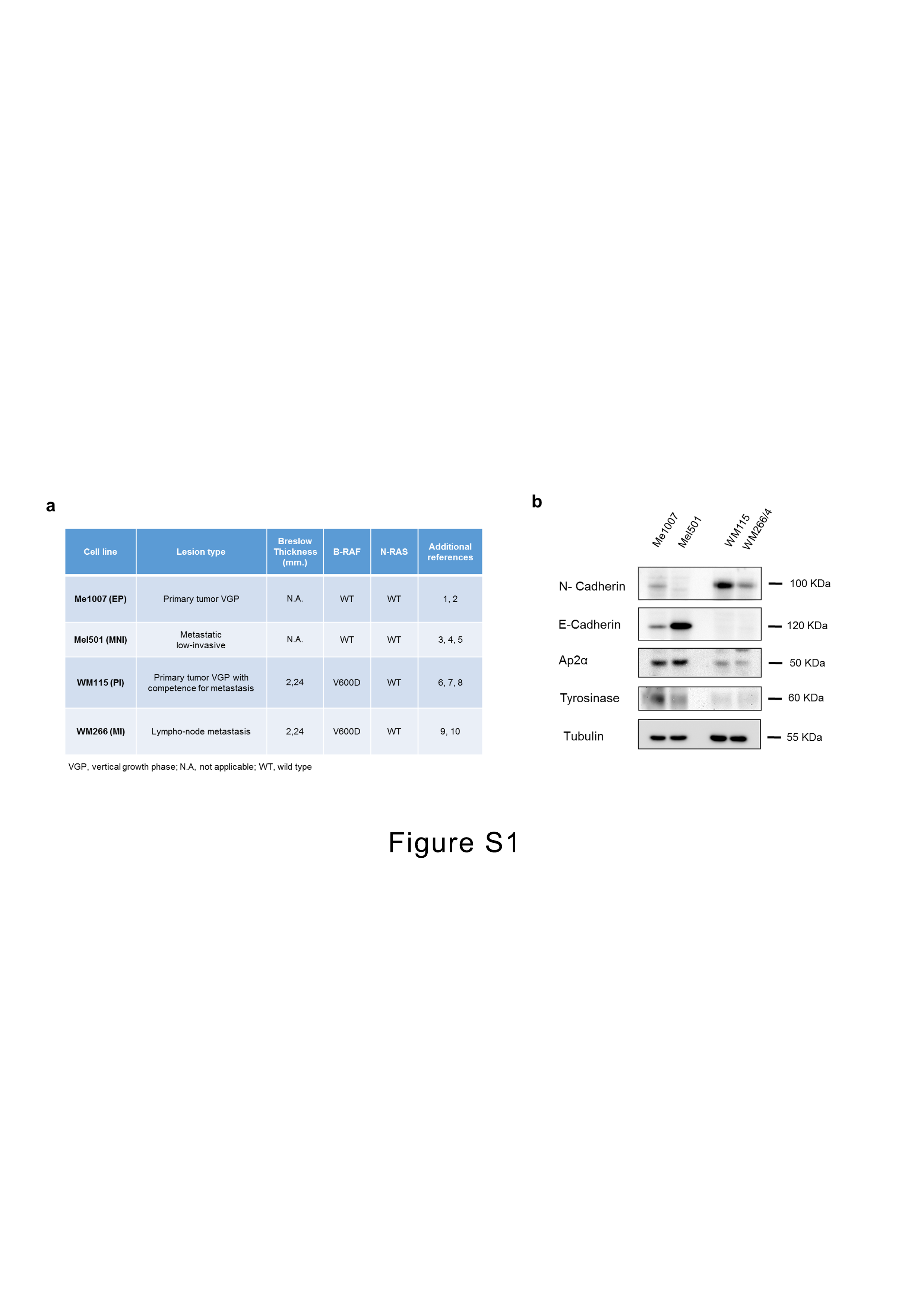

Supplement: Supplementary file 1 — Figure S1. Characterization of cell lines used in this study. a Overview table depicting classification and gene mutations. b Western blotting analysis of total cell lysates (20 μg/lane), with the indicated antibodies. (PNG 369 kb) [file 13046_2018_915_MOESM1_ESM.png]

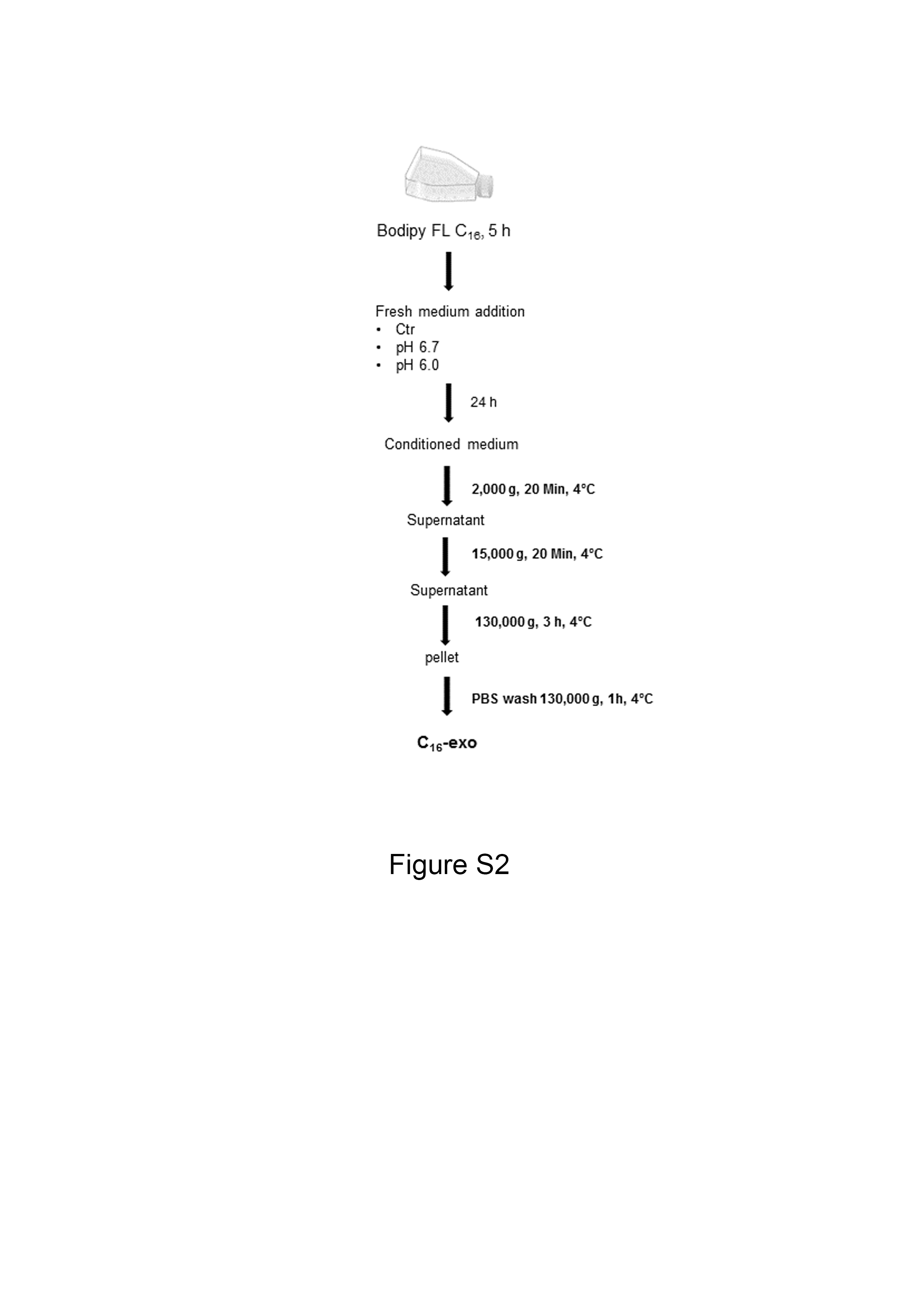

Supplement: Supplementary file 2 — Figure S2. Experimental workflow of C16-exo isolation from MNI cell culture. (PNG 271 kb) [file 13046_2018_915_MOESM2_ESM.png]

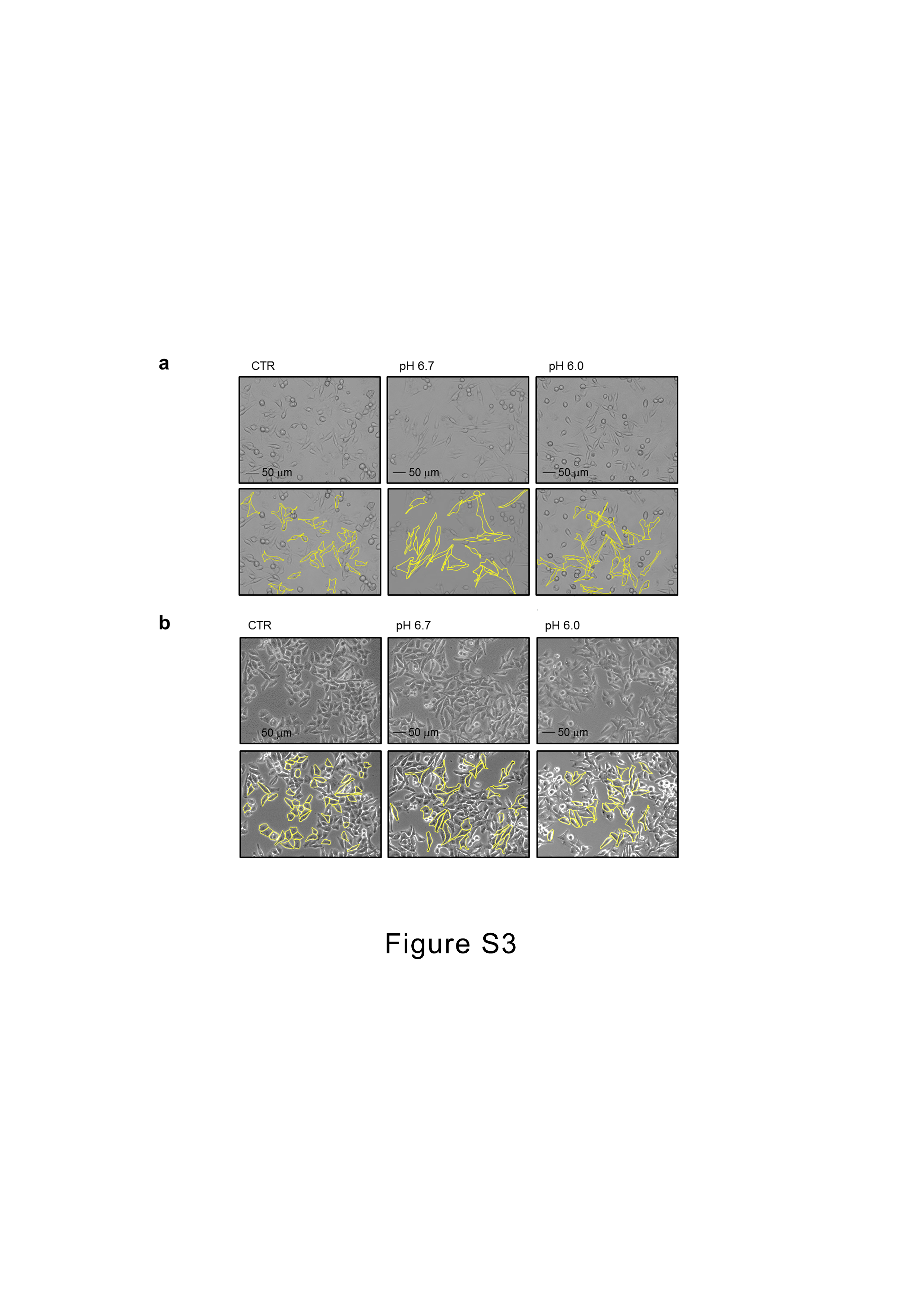

Supplement: Supplementary file 4 — Figure S3. a MNI, and b EP cells phase contrast with bounds of the selected cells (n = 30) for each condition. The lower microphotographs depict the selected cells by means of the Region of Interest (ROI, yellow lines) plugin of ImageJ. The selected cells were analyzed for elongation and perimeter by the determination of Feret’s diameter. (PNG 1662 kb) [file 13046_2018_915_MOESM4_ESM.png]

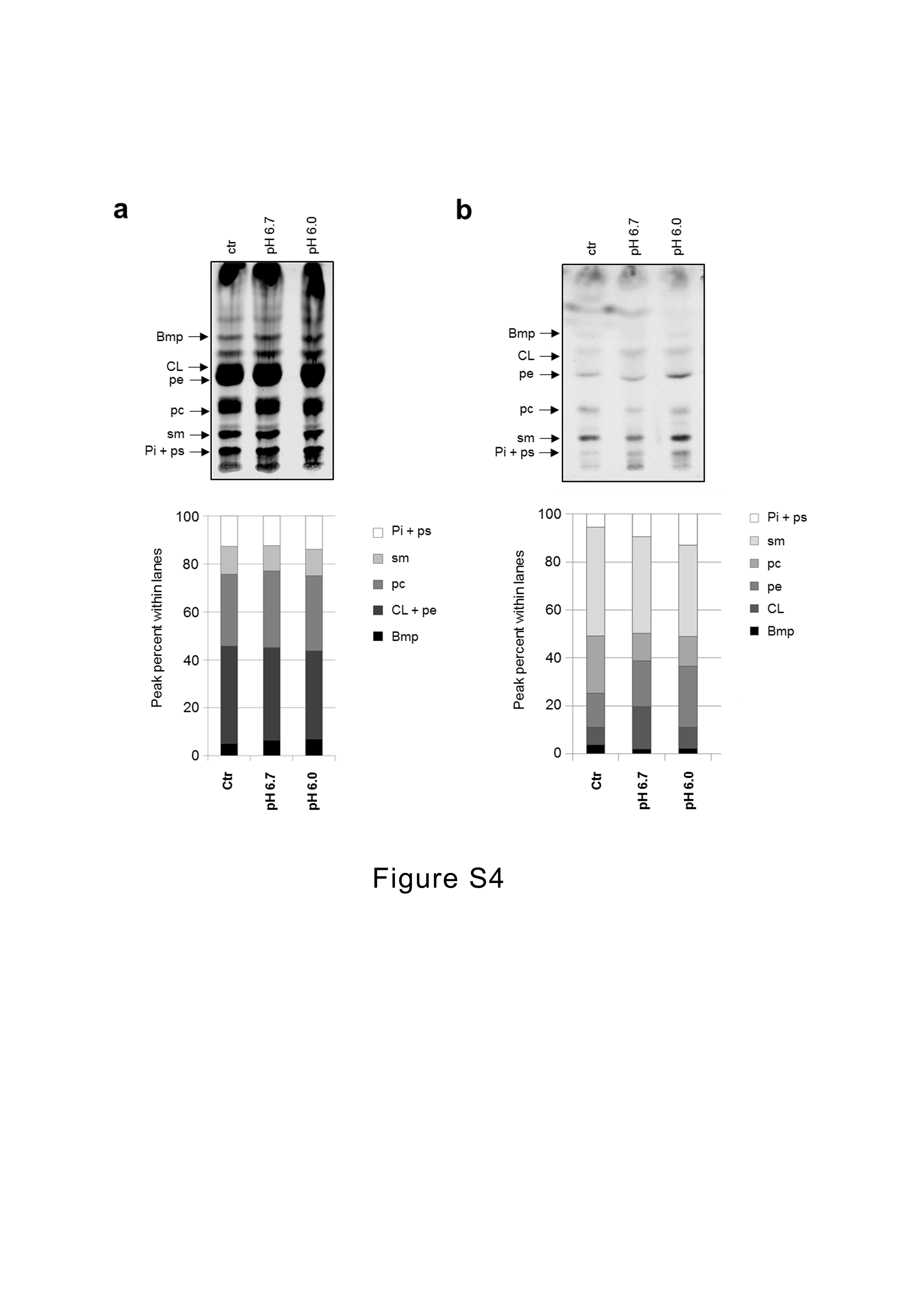

Supplement: Supplementary file 5 — Figure S4. TLC analysis of fluorescent lipids of MNI cells and exosomes. a Cells at the indicated pHs (above) and quantification values (below) of each depicted peak (arrows) as percentage value within each lane. b Exosomes at the indicated pHs (above) and quantification values (below) of each depicted peak (arrows) as percentage value within each lane. Sphingomyelin (SM), cardiolipin (CL), phosphatidylserine (PS), phosphatidylinositol (PI), phosphatidylethanolamine (PE), phosphatidylcholine (PC) and bis(monoacylglycero)phospahate (BMP). (PNG 410 kb) [file 13046_2018_915_MOESM5_ESM.png]

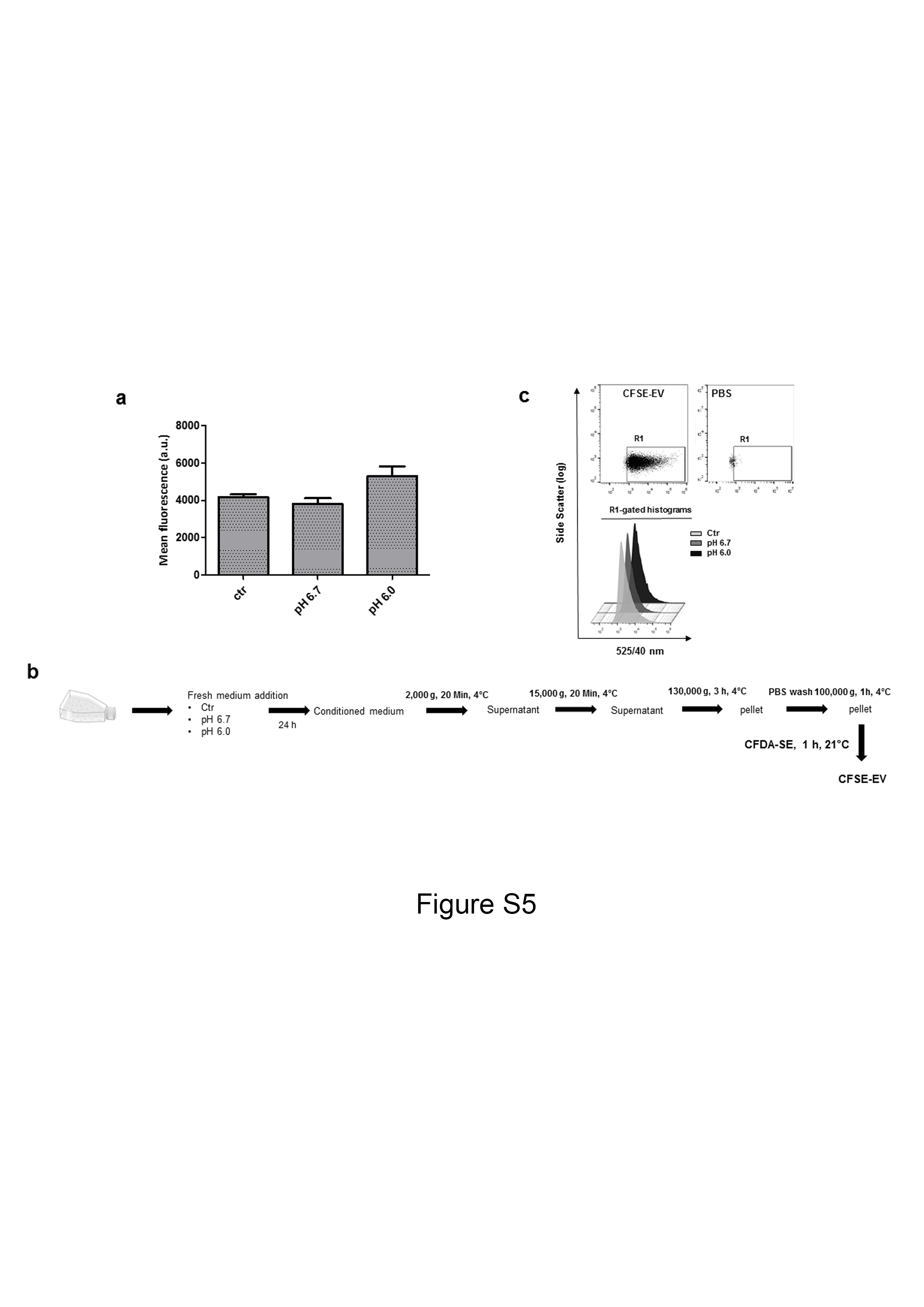

Supplement: Supplementary file 6 — Figure S5. MNI cells labeling with CFDA-SE, vesicles isolation and FACS analysis. a MNI cells were left untreated, or pH 6.7, and pH 6.0 treated for 22 h followed by 0.3 μM CFDA-SE labeling 5 min at room temperature, then FACS analyzed. Bars: mean ± S.D. (n = 3). b Workflow of vesicles isolation from MNI cells. Vesicles were labelled with (10 μM) CFDA-SE for 1 h at room temperature to obtain CFSE-EV. c FACS analysis of CFSE-EV population deriving from MNI cells cultivated at different pHs. To design the R1 region above instrument background noise only PBS was acquired. Note that no events were acquired in this region. Histograms represent the green fluorescence intensity distribution of events gated in R1 regions of CFSE population recovered from MNI cells in standard culture condition (ctr), pH 6.7 and pH 6.0. (PNG 383 kb) [file 13046_2018_915_MOESM6_ESM.png]

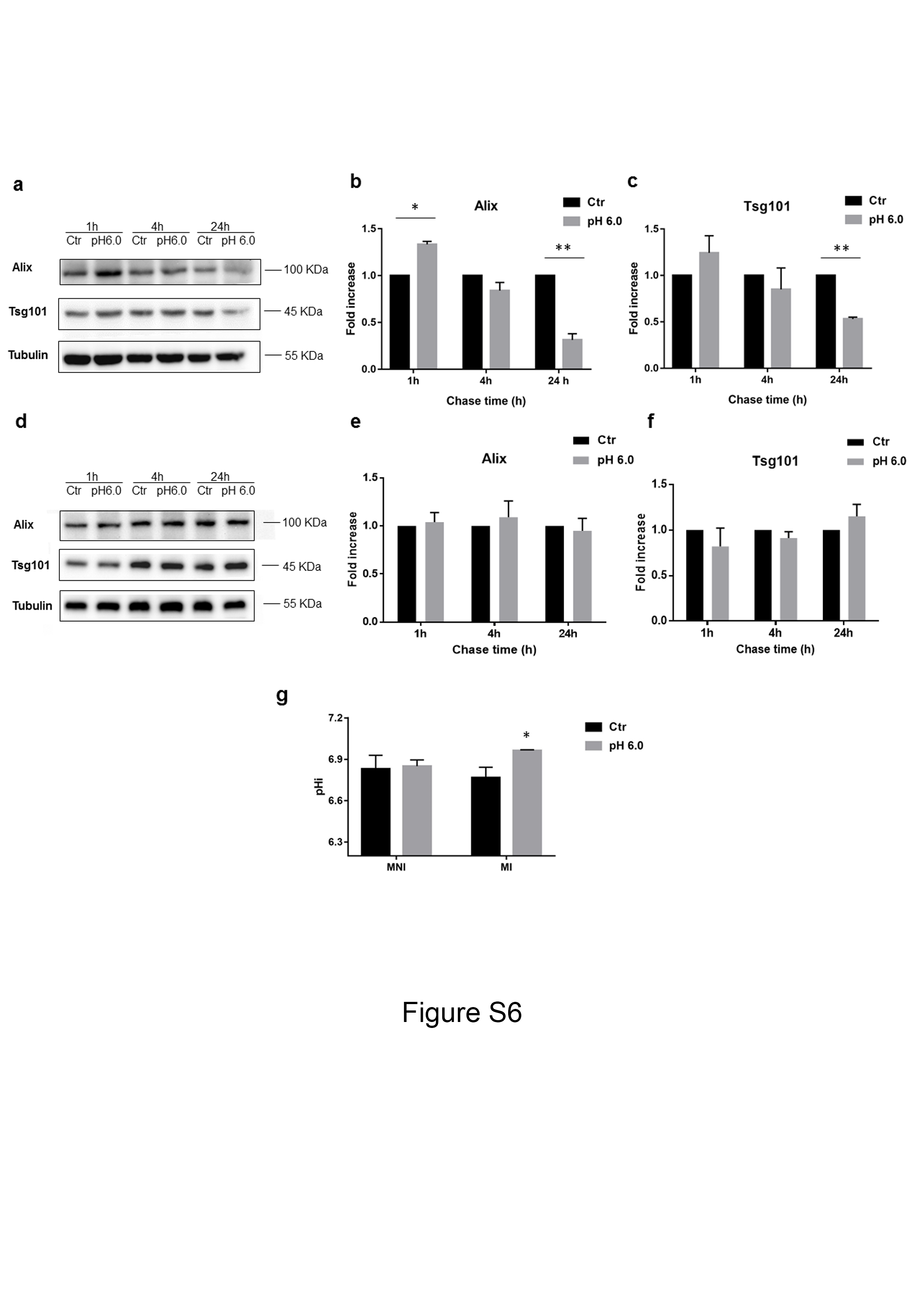

Supplement: Supplementary file 7 — Figure S6. MNI and MI intracellular expression of exosome markers and intracellular pH (pHi) evaluation a-c. MNI, and d-f MI cells were left untreated or pH 6.0 treated for the indicated times, then cells were lysed and 30 μg/lane analyzed for Alix and Tsg101 by western blot. a, d representative western blots are showed. b, e Alix, and c, f Tsg101 expression were normalized against tubulin by densitometry analysis and expressed as fold increase (n = 3),. g pHi measurement. Cells were left untreated or incubated at pH 6.0. After 24 h intracellular pH was measured with 3 μM BCECF-AM for 45 min at 37 °C. Mean ± s.d. (n = 3) *, p < 0,05 **, p < 0,01. (PNG 485 kb) [file 13046_2018_915_MOESM7_ESM.png]

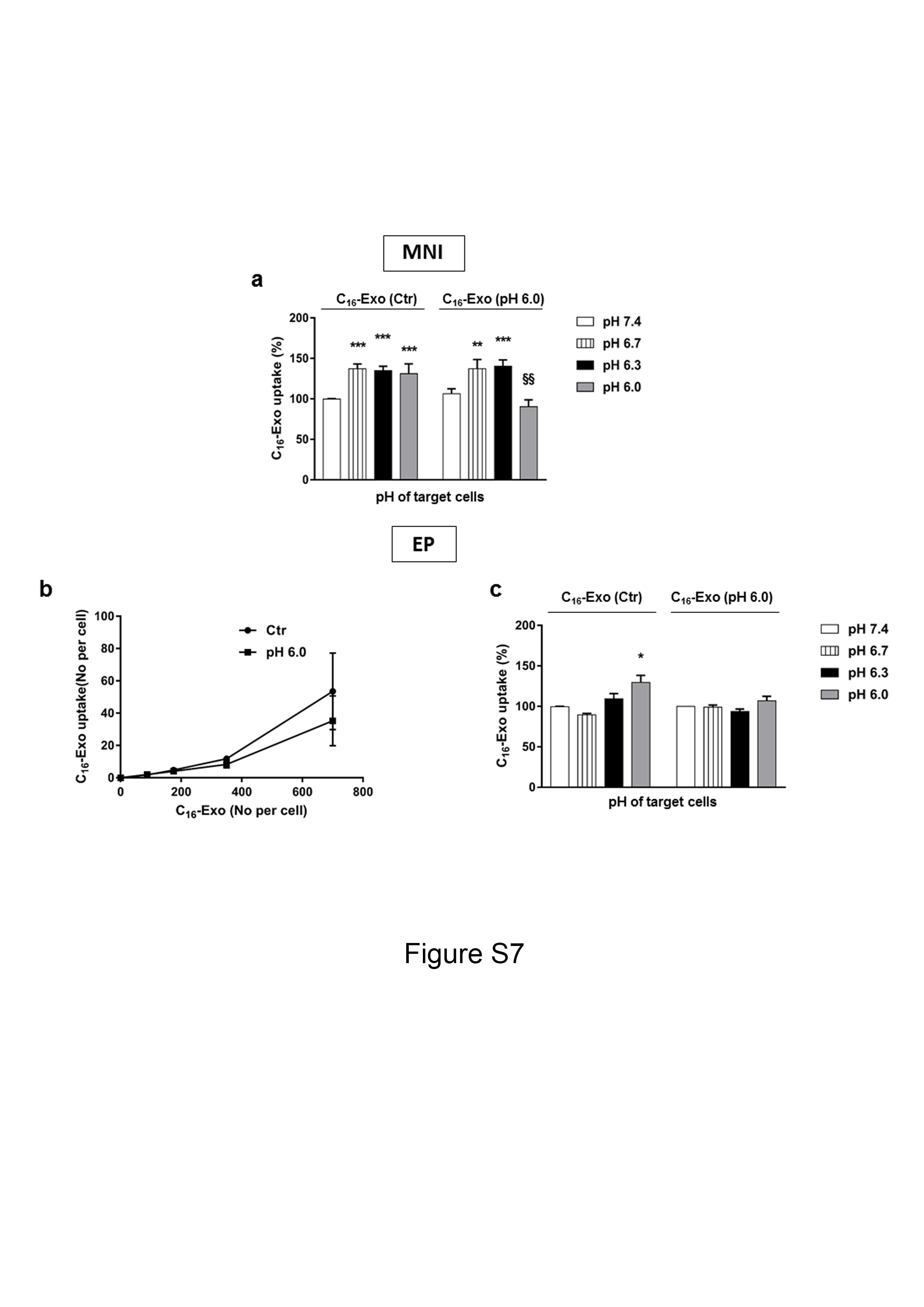

Supplement: Supplementary file 8 — Figure S7. C16-exo uptake at extracellular acid pH. 17 × 106 C16-exo (ctr) and (pH 6.0) obtained from a MNI cells and c EP cells, were incubated for 2 h at 37 °C with parental cells (0.05 × 10^6^) at the indicated pHs. The number of incorporated exosomes per cell was calculated and represented as percentage of respective control at pH 7.4. b C16-exo (ctr) and (pH 6.0) from EP cells were incubated at increasing doses with EP cells at pH 7.4 for 2 h at 37 °C. Cell fluorescence was analyzed by FACS and the number of transferred exosome calculated as described in the text. Points: mean ± S.D. (n = 3). *, p < 0,05; **, p < 0,01; ***, p < 0,005 vs respective pH 7.4 condition; §§, p < 0,01 vs C16-exo ctr at pH 7.4. (PNG 360 kb) [file 13046_2018_915_MOESM8_ESM.png]

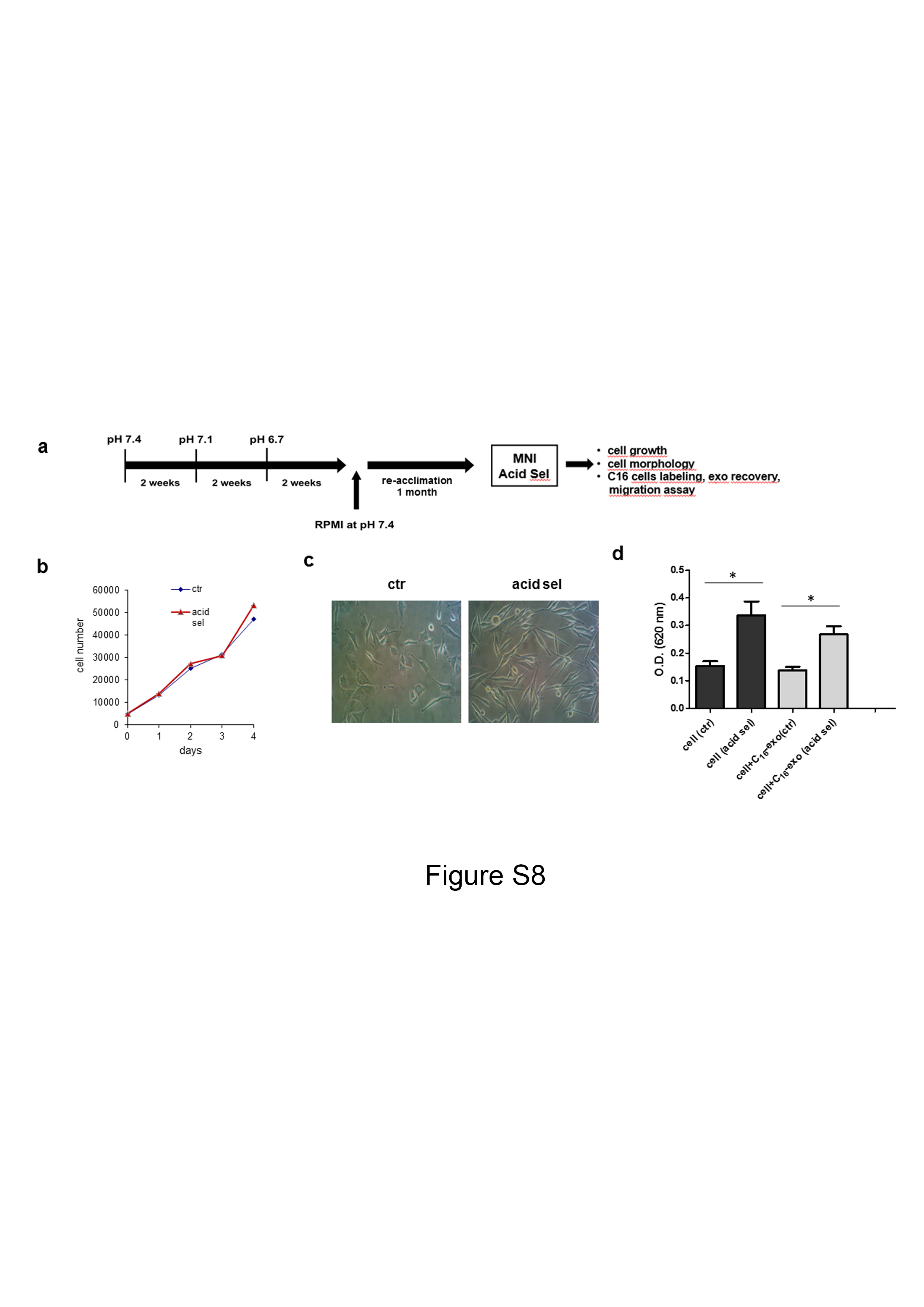

Supplement: Supplementary file 9 — Figure S8. MNI cells selection at acid pH. a Workflow of MNI selection at acid pH. At the end of acclimation cells were assayed for b growth, c morphology (magnification 10×) and d migration. MNI ctr and MNI acid sel. C16-exo were obtained as described in the manuscript, and same number (10 × 106) incubated for 72 h with MNI ctr cells. (n = 3) *, p < 0.05. (PNG 596 kb) [file 13046_2018_915_MOESM9_ESM.png]

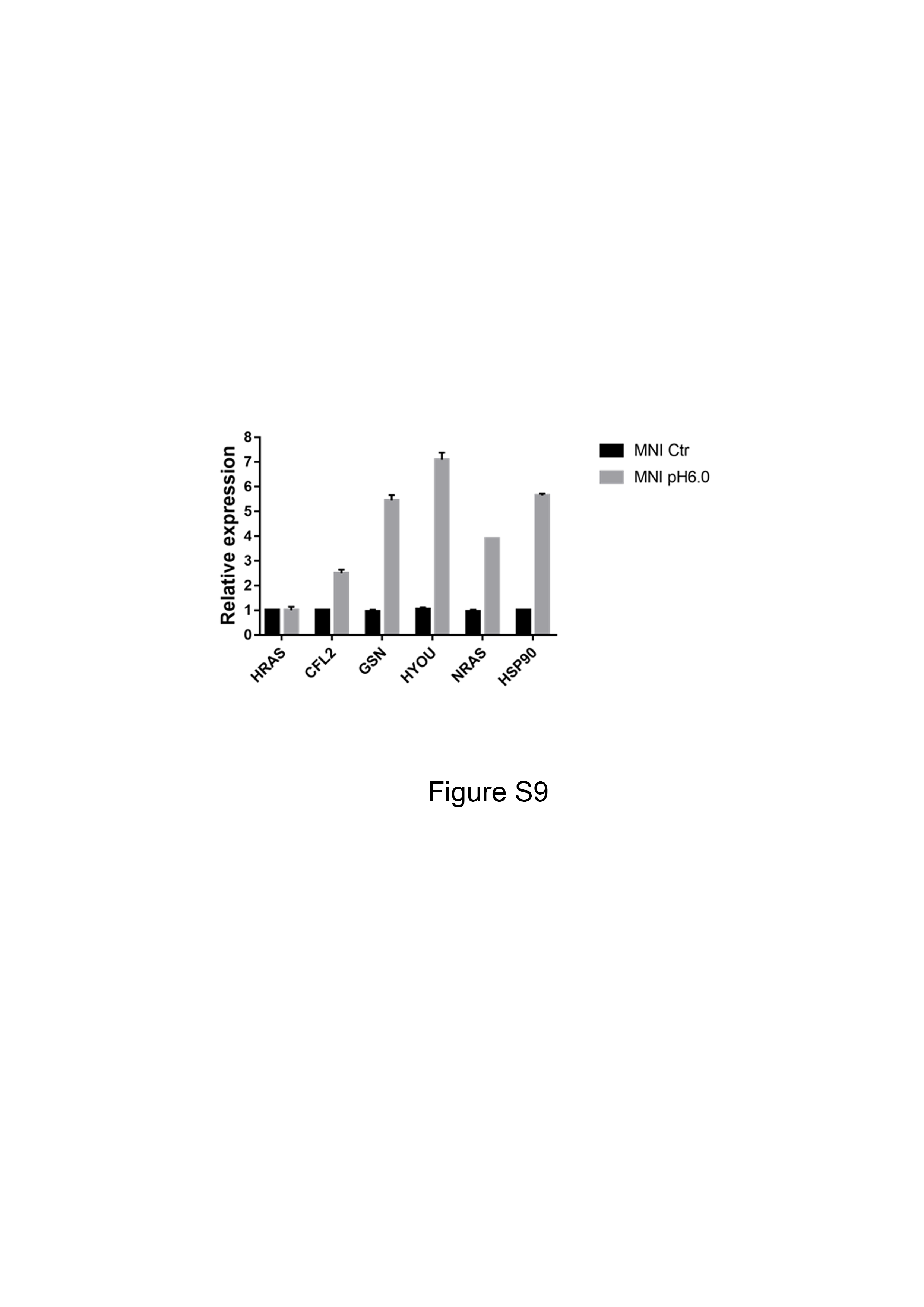

Supplement: Supplementary file 13 — Figure S9. qRT–PCR in MNI ctr and pH 6.0 treated (24 h) cells. Relative gene expression levels were normalized on GAPDH. (PNG 158 kb) [file 13046_2018_915_MOESM13_ESM.png]
