# Supplementary material for: Acidic microenvironment plays a key role in human melanoma progression through a sustained exosome mediated transfer of clinically relevant metastatic molecules
Source: J Exp Clin Cancer Res. 2018 Oct 5;37:245. doi: 10.1186/s13046-018-0915-z (PMC6173926; doi:10.1186/s13046-018-0915-z)
Supplement: Supplementary file 3 — Additional Methods. (DOCX 22 kb) [file 13046_2018_915_MOESM3_ESM.docx]

**Additional Methods**

**TLC analysis of fluorescent phospholipids**

Plates were scanned with a Typhoon Phosphorimager system (GE Healthcare Life Sciences, USA) and the fluorescence intensity of the lipid bands quantified using ImageJ software with the appropriate plugins (https://imagej.nih.gov/ij/). Non fluorescent phospholipid standards were visualized by immersing the TLC plate for 10 min in staining solution (% copper acetate and 8 % ortofosforic acid), and baked in preheated oven for 10 min at 120 °C. Plate was scanned and image analyzed with Alphaview software (Protein Simple, San Jose, CA, USA).

**Isolation of CFSE-EV, FACS analysis and quantification of C_16_-exo and CFSE-EV populations**

To obtain CFSE-EV in some experiments MNI cells were left unlabelled and cultured with exosome-depleted FBS (System Biosciences). Vesicular pellet obtained at 100,000g was resuspended with PBS and labelled with carboxylfluorescein diacetate succinimidyl-ester (CFDA-SE) (ThermoFisher Scientific) 10 µM for 1 h at 21°C.

To circumvent the risk of aspecific signal at FACS analysis due to the presence of unincorporated CFDA-SE dye, samples were diluted 1:30. The negative control was obtained by adding same amount of dye in PBS at the same condition. Samples were immediately FACS analysed.

C_16_-exo and CFSE-EV obtained from ultracentrifugation were PBS resuspended (200 µl). 2 µl were analyzed with GALLIOS cytometer (Beckman Coulter) in 180 µl PBS and additioned with 20 µl Flow-Count Fluorospheres (Beckman Coulter).

Analysis was performed by plotting fluorescence at 525/540 nm (FL1) versus log scale side scatter (SSarea) to determine the level of fluorescence threshold able to exclude the background noise. The instrument was set at flux high and at stopping gate of 2,000 Flow-Count Fluorospheres on fluorescence intensity above noise on PBS sample, and events in the correctly designed R1 region in FL1 channel were registered.

C_16_-exo or CFSE-EV total number was established according the formula: *x* = ((*y* × *a*/*b*)/*c*) × *d* where *y* = events counted at 2000 counting beads; *a* = number of counting beads in the sample; *b* = number of counting beads registered (2000); *c* = volume of sample analyzed; and *d* = total volume of exosome preparation.

To evaluate C_16_-exo and CFSE-EV dimension, commercially available size standard fluorescent beads (green fluorescent 505/515 flow cytometry sub-micron particle size reference kit) were used.

**Optiprep^TM^  gradient centrifugation**

Solutions of 40, 30, 10% iodixanol were made by mixing appropriate amounts of a homogenization buffer ( 0.25 M sucrose, 1 mM EDTA, 10 mM tris-HCl (pH 7.4)) and an iodixanol working solution (0.25 M sucrose, 6 mM EDTA, 60 mM Tris, pH 7.4 plus stock solution of Optiprep 60% (w/v), (Axis-Shield PoC, Norway).

C_16_-exo (ctr), (pH 6.7), (pH 6.0) were resuspended in PBS (260 µl), and added to 1 ml Optiprep 60% and placed at the bottom of polyallomer tube. The gradient was formed by layering 0.5 ml of 40 %, 0.5 ml of 30 %, 1,8 ml of 10% solutions on top of each other and centrifuged for 19 h at 192,000 g in a SW60 rotor (Beckman Coulter). 12 fractions of 330 µl each were collected from the top of the tube.

**Mass spectrometry analysis and data processing**

Gel slices were destained in 50 mm NH_4_CO_3_/CH_3_CN 1:1 and shrunk in acetonitrile. The acetonitrile was removed and the gel particles were dried by centrifugation under vacuum. Proteins were reduced (in 10 mm DTT, 25 mm NH_4_CO_3_, for 30 min at 56 °C), shrunk again, and alkylated (55 mm iodoacetamide, 25 mm NH_4_CO_3_, for 30 min in the dark at room temperature). Gel pieces were washed in 50 mm NH_4_CO_3_/CH_3_CN 1:1, shrunk in acetonitrile and dried by centrifugation under vacuum. In-gel digestion was performed by adding 12.5 ng/μl of trypsin (Promega, Madison, WI) in 25 mm NH_4_CO_3_ at 37 °C overnight under stirring. Supernatants were directly used for mass spectrometry analysis.

Nano-RPLC was performed using a nano-HPLC 3000 Ultimate (Dionex, Sunnyvale, CA ) connected to LTQ-XL linear ion trap (Thermo Fisher). Tryptic digests were firstly loaded on a C18 RP-precolumn (300µm i.d.x5 mm; 5μm particle size; 100 Å pore size; LC Packings-Dionex,), washed by the loading pump at 20µL/min with buffer A (5% ACN, 0.1% FA) for 5 min and then on an homemade 12 cm x 75µm- i.d. Silica PicoTip (8 ±1µm) column (NewObjective, Ringoes, NJ ) packed with C18AQ (5μm particle size; 200 Å pore size, Michrom Bioresouces Inc.) for chromatographic separation. Peptides were eluted at 0.3 µL/min along a 40 min linear gradient to 60% of buffer B (95% ACN, 0.1% FA) and electrosprayed directly into the mass spectrometer. The acquisition was performed in data-dependent Top5 method, with a minimum signal threshold of 200 counts and dynamic exclusion enabled for 30 sec.

Spectra files were analyzed by Sequest HT search engine with Proteome Discoverer 1.4 (ThermoFisher) against the Uniprot Human Reviewed Protein Database (2014 released version) and decoy database. The Carboamidomethylation of cysteines was specified as fixed modification and the oxidation of methionine was set as variable modification; mass tolerance was set to 1 Da for precursor ion and 0.4 Da for fragment ions and a maximum of two missed cleavages was allowed. The Percolator tool was used for peptide validation based on the q-value and high confidence was chosen, corresponding to a false discovery rate (FDR) ≤1% on peptide-level. Proteins were identified with a minimum of 2 peptides rank=1.

**Confocal microscopy**

Images were taken by a FV1000 confocal microscope (Olympus, Tokyo, Japan), using a (Olympus) planapo objective 60x oil A.N. 1,42. Excitation light was obtained by a Laser Dapi 408 nm for DAPI, an Argon Ion Laser (488 nm) for FITC (Alexa 488). Images recorded have an optical thickness of 0.3 mm.

Several fields were analyzed for each labeling condition, and representative images are shown.

**RNA preparation and qRT‑PCR**

RNA was isolated from cell lines using the “Total RNA Purification micro Kit” (NorgenBioteK Corp, Canada) according to the manufacturer’s protocol. Real Time quantification (qRT-PCR) of HRAS (#Hs00978050_g1), CFL2 (#Hs01071313_g1), GSN (#Hs00609272_m1), HYOU1 (#Hs00197328_m1), NRAS (#Hs00180035_m1) and HSP90AB (#Hs03043878_g1) were performed according to the TaqMan technology (Applied Biosystems, Foster City, CA, USA). GAPDH (4326317E) was used as internal control.

**Acid selection of melanoma cell line**

RPMI medium was supplemented with MES 20 mM, Mops 20mM, HEPES 20 mM, and pH was lowered each two weeks to reach pH 7.1 and pH 6.7. Then, medium was replaced with RPMI pH 7.4 for 1 month to obtain MNI acid selected cells (Acid sel).

**Western blotting** **of melanoma cell lines**

The analysis was performed according to standard procedures. Melanoma cells, were lysed, centrifuged at 10.000 x g for 10 minutes and then assayed for protein quantification. For all cell lines, 20μg of protein were suspended in Laemmli sample buffer, with freshly added 50 µM DTT, and loaded on a SDS-PAGE polyacrylamide gel. Antibodies listed below were used in accordance to the manufacturer’s instructions: Mouse monoclonal antibody to Tyrosinase (T311 #sc-20035, Santa Cruz, Dallas, TX USA), Rabbit polyclonal antibody to Ap2α (C-18 #sc-184-R, Santa Cruz, Dallas, TX USA), Mouse monoclonal antibody to E-Cadherin (36/E-Cadherin #610181, BD Transduction Laboratories™, Lexington, KY USA), Rabbit Polyclonal Antibody to N-Cadherin (#NB600-1038, Novus, Littleton, CO USA), Mouse monoclonal antibody to α-Tubulin (B-5-1-2 #T5168, SIGMA-Aldrich, Saint Louis, MO USA)

**Measurement of intracellular pH (pHi).**

Cells were left untreated or incubated at pH 6.0. After 24 h intracellular pH was measured with BCECF-AM according to Additional Ref. 11.

Briefly, cells were incubated with 3µM BCECF-AM for 45 minutes at 37 °C, washed and incubated for 10 minutes with culture medium without serum. After 2 washes in PBS, cells fluorescence were analyzed at spectrofluorometer FluoroMax-2 (Spex). Fluorescence was measured at 438-nm and 503-nm excitation, and 530-nm emission wavelengths.
