# Supplementary material for: Acidic microenvironment plays a key role in human melanoma progression through a sustained exosome mediated transfer of clinically relevant metastatic molecules
Source: J Exp Clin Cancer Res. 2018 Oct 5;37:245. doi: 10.1186/s13046-018-0915-z (PMC6173926; doi:10.1186/s13046-018-0915-z)
Supplement: Supplementary file 10 — Table S1. Identification of exosomal markers in melanoma exosomes by mass spectrometry. Table reporting the list of identified exosomal proteins, according to the Exocarta database [39], in control (ctr) or pH 6.0- exosomes from MNI cells. x, proteins identified. (DOCX 17 kb) [file 13046_2018_915_MOESM10_ESM.docx]

| **Table S1. Identification of exosomal markers in melanoma exosomes by mass spectrometry.** | | | **Condition** | |
| --- | --- | --- | --- | --- |
| **Exocarta** | **Description** | **Protein name** | **ctr** | **pH 6.0** |
| **1** | Annexin A4 (35-beta calcimedin) (Annexin IV) (Annexin-4) (Carbohydrate-binding protein p33/p41) (Chromobindin-4) (Endonexin I) (Lipocortin IV) (P32.5) (PP4-X) (Placental anticoagulant protein II) (PAP-II) (Protein II) | ANXA4_HUMAN |  | x |
| **2** | Cell division control protein 42 homolog (G25K GTP-binding protein) | CDC42_HUMAN |  | x |
| **3** | Flotillin-1 | FLOT1_HUMAN |  | x |
| **4** | Lysosome-associated membrane glycoprotein 2 (LAMP-2) (Lysosome-associated membrane protein 2) (CD107 antigen-like family member B) (LGP-96) (CD antigen CD107b) | LAMP2_HUMAN |  | x |
| **5** | Myosin-9 (Cellular myosin heavy chain, type A) (Myosin heavy chain 9) (Myosin heavy chain, non-muscle IIa) (Non-muscle myosin heavy chain A) (NMMHC-A) (Non-muscle myosin heavy chain IIa) (NMMHC II-a) (NMMHC-IIA) | MYH9_HUMAN |  | x |
| **6** | Peptidyl-prolyl cis-trans isomerase A (PPIase A) (EC 5.2.1.8) (Cyclophilin A) (Cyclosporin A-binding protein) (Rotamase A) [Cleaved into | PPIA_HUMAN |  | x |
| **7** | Ras-related protein Rab-8A (Oncogene c-mel) | RAB8A_HUMAN |  | x |
| **8** | Transitional endoplasmic reticulum ATPase (TER ATPase) (EC 3.6.4.6) (15S Mg(2+)-ATPase p97 subunit) (Valosin-containing protein) (VCP) | TERA_HUMAN | x | x |
| **9** | Alpha-actinin-4 (Non-muscle alpha-actinin 4) | ACTN4_HUMAN | x | x |
| **10** | Chloride intracellular channel protein 1 (Chloride channel ABP) (Nuclear chloride ion channel 27) (NCC27) (Regulatory nuclear chloride ion channel protein) (hRNCC) | CLIC1_HUMAN | x | x |
| **11** | Peroxiredoxin-1 (EC 1.11.1.15) (Natural killer cell-enhancing factor A) (NKEF-A) (Proliferation-associated gene protein) (PAG) (Thioredoxin peroxidase 2) (Thioredoxin-dependent peroxide reductase 2) | PRDX1_HUMAN | x | x |
| **12** | Pyruvate kinase PKM (EC 2.7.1.40) (Cytosolic thyroid hormone-binding protein) (CTHBP) (Opa-interacting protein 3) (OIP-3) (Pyruvate kinase 2/3) (Pyruvate kinase muscle isozyme) (Thyroid hormone-binding protein 1) (THBP1) (Tumor M2-PK) (p58) | KPYM_HUMAN | x | x |
| **13** | Erythrocyte band 7 integral membrane protein (Protein 7.2b) (Stomatin) | STOM_HUMAN | x | x |
| **14** | Glyceraldehyde-3-phosphate dehydrogenase (GAPDH) (EC 1.2.1.12) (Peptidyl-cysteine S-nitrosylase GAPDH) (EC 2.6.99.-) | G3P_HUMAN | x | x |
| **15** | Annexin A2 (Annexin II) (Annexin-2) (Calpactin I heavy chain) (Calpactin-1 heavy chain) (Chromobindin-8) (Lipocortin II) (Placental anticoagulant protein IV) (PAP-IV) (Protein I) (p36) | ANXA2_HUMAN | x | x |
| **16** | Annexin A6 (67 kDa calelectrin) (Annexin VI) (Annexin-6) (Calphobindin-II) (CPB-II) (Chromobindin-20) (Lipocortin VI) (Protein III) (p68) (p70) | ANXA6_HUMAN | x | x |
| **17** | Annexin A5 (Anchorin CII) (Annexin V) (Annexin-5) (Calphobindin I) (CBP-I) (Endonexin II) (Lipocortin V) (Placental anticoagulant protein 4) (PP4) (Placental anticoagulant protein I) (PAP-I) (Thromboplastin inhibitor) (Vascular anticoagulant-alpha) (VAC-alpha) | ANXA5_HUMAN | x | x |
| **18** | Actin, cytoplasmic 1 (Beta-actin) [Cleaved into | ACTB_HUMAN | x | x |
| **19** | Ras-related protein Rap-1b (GTP-binding protein smg p21B) | RAP1B_HUMAN | x | x |
| **20** | Ras-related protein Rab-1A (YPT1-related protein) | RAB1A_HUMAN | x | x |
| **21** | L-lactate dehydrogenase B chain (LDH-B) (EC 1.1.1.27) (LDH heart subunit) (LDH-H) (Renal carcinoma antigen NY-REN-46) | LDHB_HUMAN | x | x |
| **22** | Ras-related protein Rab-7a | RAB7A_HUMAN | x | x |
| **23** | CD81 antigen (26 kDa cell surface protein TAPA-1) (Target of the antiproliferative antibody 1) (Tetraspanin-28) (Tspan-28) (CD antigen CD81) | CD81_HUMAN | x | x |
| **24** | Guanine nucleotide-binding protein G(i) subunit alpha-2 (Adenylate cyclase-inhibiting G alpha protein) | GNAI2_HUMAN | x | x |
| **25** | CD82 antigen (C33 antigen) (IA4) (Inducible membrane protein R2) (Metastasis suppressor Kangai-1) (Suppressor of tumorigenicity 6 protein) (Tetraspanin-27) (Tspan-27) (CD antigen CD82) | CD82_HUMAN | x |  |
| **26** | 60S ribosomal protein L12 (Large ribosomal subunit protein uL11) | RL12_HUMAN | x |  |
| **27** | HLA class I histocompatibility antigen, A-1 alpha chain (MHC class I antigen A*1) | 1A01_HUMAN | x |  |
| **28** | Ras-related protein Rab-5C (L1880) (RAB5L) | RAB5C_HUMAN | x |  |
