# Supplementary material for: Acidic microenvironment plays a key role in human melanoma progression through a sustained exosome mediated transfer of clinically relevant metastatic molecules
Source: J Exp Clin Cancer Res. 2018 Oct 5;37:245. doi: 10.1186/s13046-018-0915-z (PMC6173926; doi:10.1186/s13046-018-0915-z)
Supplement: Supplementary file 11 — Table S2. List of exosomal proteins from MNI cells identified by mass-spectrometry and found upregulated and down-regulated at pH 6.0, or equally expressed in control condition. In case of proteins specific for each condition emPAInorm value is reported. In case of proteins found in both conditions emPAInorm ratio (ρ) is shown. (DOCX 59 kb) [file 13046_2018_915_MOESM11_ESM.docx]

**Table S2.** List of exosomal proteins from MNI cells identified by mass-spectrometry and found upregulated and down-regulated at pH 6.0 , or equally expressed in control condition.

| **pH 6.0 up regulated** | | | | |  |  | |  |  |  |  | |
| --- | --- | --- | --- | --- | --- | --- | --- | --- | --- | --- | --- | --- |
|  | | | | |  |  | |  | **emPAInorm ratio** |  |  | |
| **ID** | **emPAInorm** | |  | | **ID** | **emPAInorm** | | | **pH6.0/ctr** | **Gene name** | **description** |  |
| P11021 | 0.348164707 | | pH6 | |  |  |  | | pH6 specific | GRP78_HUMAN | 78 kDa glucose-regulated protein (GRP-78) (Endoplasmic reticulum lumenal Ca(2+)-binding protein grp78) (Heat shock 70 kDa protein 5) (Immunoglobulin heavy chain-binding protein) (BiP) |  |
| P18085 | 0.260531706 | | pH6 | |  |  |  | | pH6 specific | ARF4_HUMAN | ADP-ribosylation factor 4 |  |
| P84085 | 0.260531706 | | pH6 | |  |  |  | | pH6 specific | ARF5_HUMAN | ADP-ribosylation factor 5 |  |
| P62330 | 0.306873798 | | pH6 | |  |  |  | | pH6 specific | ARF6_HUMAN | ADP-ribosylation factor 6 |  |
| P49588 | 0.276523365 | | pH6 | |  |  |  | | pH6 specific | SYAC_HUMAN | Alanine--tRNA ligase, cytoplasmic (EC 6.1.1.7) (Alanyl-tRNA synthetase) (AlaRS) (Renal carcinoma antigen NY-REN-42) |  |
| P09525 | 0.081733463 | | pH6 | |  |  |  | | pH6 specific | ANXA4_HUMAN | Annexin A4 (35-beta calcimedin) (Annexin IV) (Annexin-4) (Carbohydrate-binding protein p33/p41) (Chromobindin-4) (Endonexin I) (Lipocortin IV) (P32.5) (PP4-X) (Placental anticoagulant protein II) (PAP-II) (Protein II) |  |
| Q4KMQ2 | 0.174721684 | | pH6 | |  |  |  | | pH6 specific | ANO6_HUMAN | Anoctamin-6 (Small-conductance calcium-activated nonselective cation channel) (SCAN channel) (Transmembrane protein 16F) |  |
| P11586 | 0.092681158 | | pH6 | |  |  |  | | pH6 specific | C1TC_HUMAN | C-1-tetrahydrofolate synthase, cytoplasmic (C1-THF synthase) [Cleaved into |  |
| P16152 | 0.345803995 | | pH6 | |  |  |  | | pH6 specific | CBR1_HUMAN | Carbonyl reductase [NADPH] 1 (EC 1.1.1.184) (15-hydroxyprostaglandin dehydrogenase [NADP(+)]) (EC 1.1.1.197) (NADPH-dependent carbonyl reductase 1) (Prostaglandin 9-ketoreductase) (Prostaglandin-E(2) 9-reductase) (EC 1.1.1.189) (Short chain dehydrogenase/reductase family 21C member 1) |  |
| O60716 | 0.424231823 | | pH6 | |  |  |  | | pH6 specific | CTND1_HUMAN | Catenin delta-1 (Cadherin-associated Src substrate) (CAS) (p120 catenin) (p120(ctn)) (p120(cas)) |  |
| P13987 | 0.380397373 | | pH6 | |  |  |  | | pH6 specific | CD59_HUMAN | CD59 glycoprotein (1F5 antigen) (20 kDa homologous restriction factor) (HRF-20) (HRF20) (MAC-inhibitory protein) (MAC-IP) (MEM43 antigen) (Membrane attack complex inhibition factor) (MACIF) (Membrane inhibitor of reactive lysis) (MIRL) (Protectin) (CD antigen CD59) |  |
| P60953 | 0.533742135 | | pH6 | |  |  |  | | pH6 specific | CDC42_HUMAN | Cell division control protein 42 homolog (G25K GTP-binding protein) |  |
| P23528 | 0.556144515 | | pH6 | |  |  |  | | pH6 specific | COF1_HUMAN | Cofilin-1 (18 kDa phosphoprotein) (p18) (Cofilin, non-muscle isoform) |  |
| Q9Y281 | 0.186887261 | | pH6 | |  |  |  | | pH6 specific | COF2_HUMAN | Cofilin-2 (Cofilin, muscle isoform) |  |
| P15924 | 0.041939421 | | pH6 | |  |  |  | | pH6 specific | DESP_HUMAN | Desmoplakin (DP) (250/210 kDa paraneoplastic pemphigus antigen) |  |
| Q86T65 | 0.061888805 | | pH6 | |  |  |  | | pH6 specific | DAAM2_HUMAN | Disheveled-associated activator of morphogenesis 2 |  |
| O60884 | 0.09396292 | | pH6 | |  |  |  | | pH6 specific | DNJA2_HUMAN | DnaJ homolog subfamily A member 2 (Cell cycle progression restoration gene 3 protein) (Dnj3) (Dj3) (HIRA-interacting protein 4) (Renal carcinoma antigen NY-REN-14) |  |
| P14625 | 0.167802911 | | pH6 | |  |  |  | | pH6 specific | ENPL_HUMAN | Endoplasmin (94 kDa glucose-regulated protein) (GRP-94) (Heat shock protein 90 kDa beta member 1) (Tumor rejection antigen 1) (gp96 homolog) |  |
| P55060 | 0.058558564 | | pH6 | |  |  |  | | pH6 specific | XPO2_HUMAN | Exportin-2 (Exp2) (Cellular apoptosis susceptibility protein) (Chromosome segregation 1-like protein) (Importin-alpha re-exporter) |  |
| O75955 | 0.096832573 | | pH6 | |  |  |  | | pH6 specific | FLOT1_HUMAN | Flotillin-1 |  |
| P06396 | 0.327947678 | | pH6 | |  |  |  | | pH6 specific | GELS_HUMAN | Gelsolin (AGEL) (Actin-depolymerizing factor) (ADF) (Brevin) |  |
| Q06210 | 0.220149303 | | pH6 | |  |  |  | | pH6 specific | GFPT1_HUMAN | Glutamine--fructose-6-phosphate aminotransferase [isomerizing] 1 (EC 2.6.1.16) (D-fructose-6-phosphate amidotransferase 1) (Glutamine |  |
| P11216 | 0.205288085 | | pH6 | |  |  |  | | pH6 specific | PYGB_HUMAN | Glycogen phosphorylase, brain form (EC 2.4.1.1) |  |
| P01112 | 0.172275906 | | pH6 | |  |  |  | | pH6 specific | RASH_HUMAN | GTPase HRas (H-Ras-1) (Ha-Ras) (Transforming protein p21) (c-H-ras) (p21ras) [Cleaved into |  |
| P01111 | 0.265767125 | | pH6 | |  |  |  | | pH6 specific | RASN_HUMAN | GTPase NRas (Transforming protein N-Ras) |  |
| P34931 | 0.168235561 | | pH6 | |  |  |  | | pH6 specific | HS71L_HUMAN | Heat shock 70 kDa protein 1-like (Heat shock 70 kDa protein 1L) (Heat shock 70 kDa protein 1-Hom) (HSP70-Hom) |  |
| P34932 | 0.13628832 | | pH6 | |  |  |  | | pH6 specific | HSP74_HUMAN | Heat shock 70 kDa protein 4 (HSP70RY) (Heat shock 70-related protein APG-2) |  |
| Q14568 | 0.335218611 | | pH6 | |  |  |  | | pH6 specific | HS902_HUMAN | Heat shock protein HSP 90-alpha A2 (Heat shock 90 kDa protein 1 alpha-like 3) (Heat shock protein HSP 90-alpha A2 pseudogene) |  |
| P04439 | 0.320440821 | | pH6 | |  |  |  | | pH6 specific | 1A03_HUMAN | HLA class I histocompatibility antigen, A-3 alpha chain (MHC class I antigen A*3) |  |
| P16188 | 0.156011862 | | pH6 | |  |  |  | | pH6 specific | 1A30_HUMAN | HLA class I histocompatibility antigen, A-30 alpha chain (MHC class I antigen A*30) |  |
| Q9Y4L1 | 0.191168123 | | pH6 | |  |  |  | | pH6 specific | HYOU1_HUMAN | Hypoxia up-regulated protein 1 (150 kDa oxygen-regulated protein) (ORP-150) (170 kDa glucose-regulated protein) (GRP-170) |  |
| Q14974 | 0.088284383 | | pH6 | |  |  |  | | pH6 specific | IMB1_HUMAN | Importin subunit beta-1 (Importin-90) (Karyopherin subunit beta-1) (Nuclear factor p97) (Pore targeting complex 97 kDa subunit) (PTAC97) |  |
| P05362 | 0.235061878 | | pH6 | |  |  |  | | pH6 specific | ICAM1_HUMAN | Intercellular adhesion molecule 1 (ICAM-1) (Major group rhinovirus receptor) (CD antigen CD54) |  |
| P13473 | 0.465031135 | | pH6 | |  |  |  | | pH6 specific | LAMP2_HUMAN | Lysosome-associated membrane glycoprotein 2 (LAMP-2) (Lysosome-associated membrane protein 2) (CD107 antigen-like family member B) (LGP-96) (CD antigen CD107b) |  |
| Q7Z304 | 0.108592469 | | pH6 | |  |  |  | | pH6 specific | MAMC2_HUMAN | MAM domain-containing protein 2 (MAM domain-containing proteoglycan) (Mamcan) |  |
| O00339 | 0.158505922 | | pH6 | |  |  |  | | pH6 specific | MATN2_HUMAN | Matrilin-2 |  |
| P35580 | 0.160209782 | | pH6 | |  |  |  | | pH6 specific | MYH10_HUMAN | Myosin-10 (Cellular myosin heavy chain, type B) (Myosin heavy chain 10) (Myosin heavy chain, non-muscle IIb) (Non-muscle myosin heavy chain B) (NMMHC-B) (Non-muscle myosin heavy chain IIb) (NMMHC II-b) (NMMHC-IIB) |  |
| P35579 | 1.908334913 | | pH6 | |  |  |  | | pH6 specific | MYH9_HUMAN | Myosin-9 (Cellular myosin heavy chain, type A) (Myosin heavy chain 9) (Myosin heavy chain, non-muscle IIa) (Non-muscle myosin heavy chain A) (NMMHC-A) (Non-muscle myosin heavy chain IIa) (NMMHC II-a) (NMMHC-IIA) |  |
| P29966 | 1.056000007 | | pH6 | |  |  |  | | pH6 specific | MARCS_HUMAN | Myristoylated alanine-rich C-kinase substrate (MARCKS) (Protein kinase C substrate, 80 kDa protein, light chain) (80K-L protein) (PKCSL) |  |
| Q14697 | 0.344124453 | | pH6 | |  |  |  | | pH6 specific | GANAB_HUMAN | Neutral alpha-glucosidase AB (EC 3.2.1.84) (Alpha-glucosidase 2) (Glucosidase II subunit alpha) |  |
| P62937 | 0.284846103 | | pH6 | |  |  |  | | pH6 specific | PPIA_HUMAN | Peptidyl-prolyl cis-trans isomerase A (PPIase A) (EC 5.2.1.8) (Cyclophilin A) (Cyclosporin A-binding protein) (Rotamase A) [Cleaved into |  |
| P30086 | 0.339205937 | | pH6 | |  |  |  | | pH6 specific | PEBP1_HUMAN | Phosphatidylethanolamine-binding protein 1 (PEBP-1) (HCNPpp) (Neuropolypeptide h3) (Prostatic-binding protein) (Raf kinase inhibitor protein) (RKIP) [Cleaved into |  |
| O75340 | 0.407741833 | | pH6 | |  |  |  | | pH6 specific | PDCD6_HUMAN | Programmed cell death protein 6 (Apoptosis-linked gene 2 protein) (Probable calcium-binding protein ALG-2) |  |
| Q12884 | 0.385700125 | | pH6 | |  |  |  | | pH6 specific | SEPR_HUMAN | Prolyl endopeptidase FAP (EC 3.4.21.26) (170 kDa melanoma membrane-bound gelatinase) (Dipeptidyl peptidase FAP) (EC 3.4.14.5) (Fibroblast activation protein alpha) (FAPalpha) (Gelatine degradation protease FAP) (EC 3.4.21.-) (Integral membrane serine protease) (Post-proline cleaving enzyme) (Serine integral membrane protease) (SIMP) (Surface-expressed protease) (Seprase) [Cleaved into |  |
| P61106 | 0.207408998 | | pH6 | |  |  |  | | pH6 specific | RAB14_HUMAN | Ras-related protein Rab-14 |  |
| P61019 | 0.302200073 | | pH6 | |  |  |  | | pH6 specific | RAB2A_HUMAN | Ras-related protein Rab-2A |  |
| P61006 | 0.119380415 | | pH6 | |  |  |  | | pH6 specific | RAB8A_HUMAN | Ras-related protein Rab-8A (Oncogene c-mel) |  |
| Q92930 | 0.114222602 | | pH6 | |  |  |  | | pH6 specific | RAB8B_HUMAN | Ras-related protein Rab-8B |  |
| P11233 | 0.661440676 | | pH6 | |  |  |  | | pH6 specific | RALA_HUMAN | Ras-related protein Ral-A |  |
| P61225 | 0.376787226 | | pH6 | |  |  |  | | pH6 specific | RAP2B_HUMAN | Ras-related protein Rap-2b |  |
| Q12913 | 0.16666387 | | pH6 | |  |  |  | | pH6 specific | PTPRJ_HUMAN | Receptor-type tyrosine-protein phosphatase eta (Protein-tyrosine phosphatase eta) (R-PTP-eta) (EC 3.1.3.48) (Density-enhanced phosphatase 1) (DEP-1) (HPTP eta) (Protein-tyrosine phosphatase receptor type J) (R-PTP-J) (CD antigen CD148) |  |
| P08134 | 0.148979282 | | pH6 | |  |  |  | | pH6 specific | RHOC_HUMAN | Rho-related GTP-binding protein RhoC (Rho cDNA clone 9) (h9) |  |
| O75044 | 0.256709032 | | pH6 | |  |  |  | | pH6 specific | SRGP2_HUMAN | SLIT-ROBO Rho GTPase-activating protein 2 (srGAP2) (Formin-binding protein 2) (Rho GTPase-activating protein 34) |  |
| P13637 | 0.353015559 | | pH6 | |  |  |  | | pH6 specific | AT1A3_HUMAN | Sodium/potassium-transporting ATPase subunit alpha-3 (Na(+)/K(+) ATPase alpha-3 subunit) (EC 3.6.3.9) (Na(+)/K(+) ATPase alpha(III) subunit) (Sodium pump subunit alpha-3) |  |
| Q01082 | 0.056326555 | | pH6 | |  |  |  | | pH6 specific | SPTB2_HUMAN | Spectrin beta chain, non-erythrocytic 1 (Beta-II spectrin) (Fodrin beta chain) (Spectrin, non-erythroid beta chain 1) |  |
| Q7KZF4 | 0.210679971 | | pH6 | |  |  |  | | pH6 specific | SND1_HUMAN | Staphylococcal nuclease domain-containing protein 1 (100 kDa coactivator) (EBNA2 coactivator p100) (Tudor domain-containing protein 11) (p100 co-activator) |  |
| P07996 | 0.050694338 | | pH6 | |  |  |  | | pH6 specific | TSP1_HUMAN | Thrombospondin-1 |  |
| Q13885 | 0.411971975 | | pH6 | |  |  |  | | pH6 specific | TBB2A_HUMAN | Tubulin beta-2A chain (Tubulin beta class IIa) |  |
| Q9BUF5 | 0.135260284 | | pH6 | |  |  |  | | pH6 specific | TBB6_HUMAN | Tubulin beta-6 chain (Tubulin beta class V) |  |
| Q9Y4I1 | 0.070779458 | | pH6 | |  |  |  | | pH6 specific | MYO5A_HUMAN | Unconventional myosin-Va (Dilute myosin heavy chain, non-muscle) (Myosin heavy chain 12) (Myosin-12) (Myoxin) |  |
| P18206 | 0.064212856 | | pH6 | |  |  |  | | pH6 specific | VINC_HUMAN | Vinculin (Metavinculin) (MV) |  |
| P55072 | 0.526648247 | | pH6 | | P55072 | 0.038165 | pH7 | | 13.79908092 | TERA_HUMAN | Transitional endoplasmic reticulum ATPase (TER ATPase) (EC 3.6.4.6) (15S Mg(2+)-ATPase p97 subunit) (Valosin-containing protein) (VCP) |  |
| O15031 | 0.412617002 | | pH6 | | O15031 | 0.033257 | pH7 | | 12.40688249 | PLXB2_HUMAN | Plexin-B2 (MM1) |  |
| O00159 | 0.422254629 | | pH6 | | O00159 | 0.036783 | pH7 | | 11.47961129 | MYO1C_HUMAN | Unconventional myosin-Ic (Myosin I beta) (MMI-beta) (MMIb) |  |
| P02768 | 0.355791444 | | pH6 | | P02768 | 0.063627 | pH7 | | 5.591866317 | ALBU_HUMAN | Serum albumin |  |
| O43707 | 0.567962937 | | pH6 | | O43707 | 0.160279 | pH7 | | 3.543596769 | ACTN4_HUMAN | Alpha-actinin-4 (Non-muscle alpha-actinin 4) |  |
| P46940 | 0.288114116 | | pH6 | | P46940 | 0.091155 | pH7 | | 3.16069861 | IQGA1_HUMAN | Ras GTPase-activating-like protein IQGAP1 (p195) |  |
| P13639 | 0.438779727 | | pH6 | | P13639 | 0.147398 | pH7 | | 2.976833766 | EF2_HUMAN | Elongation factor 2 (EF-2) |  |
| P12814 | 0.370373707 | | pH6 | | P12814 | 0.15688 | pH7 | | 2.36086799 | ACTN1_HUMAN | Alpha-actinin-1 (Alpha-actinin cytoskeletal isoform) (F-actin cross-linking protein) (Non-muscle alpha-actinin-1) |  |
| O00299 | 1.045347622 | | pH6 | | O00299 | 0.451683 | pH7 | | 2.314340493 | CLIC1_HUMAN | Chloride intracellular channel protein 1 (Chloride channel ABP) (Nuclear chloride ion channel 27) (NCC27) (Regulatory nuclear chloride ion channel protein) (hRNCC) |  |
| Q9Y490 | 0.419166447 | | pH6 | | Q9Y490 | 0.192018 | pH7 | | 2.182958393 | TLN1_HUMAN | Talin-1 |  |
| P02786 | 0.381548236 | | pH6 | | P02786 | 0.181708 | pH7 | | 2.099790609 | TFR1_HUMAN | Transferrin receptor protein 1 (TR) (TfR) (TfR1) (Trfr) (T9) (p90) (CD antigen CD71) [Cleaved into |  |
| P49327 | 0.161155734 | | pH6 | | P49327 | 0.082071 | pH7 | | 1.963604689 | FAS_HUMAN | Fatty acid synthase (EC 2.3.1.85) [Includes |  |
| O60486 | 0.241409464 | | pH6 | | O60486 | 0.126929 | pH7 | | 1.901929347 | PLXC1_HUMAN | Plexin-C1 (Virus-encoded semaphorin protein receptor) (CD antigen CD232) |  |
| Q06830 | 0.344124453 | | pH6 | | Q06830 | 0.188924 | pH7 | | 1.821495634 | PRDX1_HUMAN | Peroxiredoxin-1 (EC 1.11.1.15) (Natural killer cell-enhancing factor A) (NKEF-A) (Proliferation-associated gene protein) (PAG) (Thioredoxin peroxidase 2) (Thioredoxin-dependent peroxide reductase 2) |  |
| Q08380 | 1.532616095 | | pH6 | | Q08380 | 0.850567 | pH7 | | 1.801876105 | LG3BP_HUMAN | Galectin-3-binding protein (Basement membrane autoantigen p105) (Lectin galactoside-binding soluble 3-binding protein) (Mac-2-binding protein) (MAC2BP) (Mac-2 BP) (Tumor-associated antigen 90K) |  |
| P68104 | 0.505191678 | | pH6 | | P68104 | 0.282068 | pH7 | | 1.791028918 | EF1A1_HUMAN | Elongation factor 1-alpha 1 (EF-1-alpha-1) (Elongation factor Tu) (EF-Tu) (Eukaryotic elongation factor 1 A-1) (eEF1A-1) (Leukocyte receptor cluster member 7) |  |
| P06733 | 0.974125582 | | pH6 | | P06733 | 0.557664 | pH7 | | 1.746796243 | ENOA_HUMAN | Alpha-enolase (EC 4.2.1.11) (2-phospho-D-glycerate hydro-lyase) (C-myc promoter-binding protein) (Enolase 1) (MBP-1) (MPB-1) (Non-neural enolase) (NNE) (Phosphopyruvate hydratase) (Plasminogen-binding protein) |  |
| P07900 | 0.46338504 | | pH6 | | P07900 | 0.266475 | pH7 | | 1.738946613 | HS90A_HUMAN | Heat shock protein HSP 90-alpha (Heat shock 86 kDa) (HSP 86) (HSP86) (Lipopolysaccharide-associated protein 2) (LAP-2) (LPS-associated protein 2) (Renal carcinoma antigen NY-REN-38) |  |
| Q9NZM1 | 0.180449005 | | pH6 | | Q9NZM1 | 0.103938 | pH7 | | 1.736119379 | MYOF_HUMAN | Myoferlin (Fer-1-like protein 3) |  |
| P16070 | 0.283010823 | | pH6 | | P16070 | 0.163827 | pH7 | | 1.72749338 | CD44_HUMAN | CD44 antigen (CDw44) (Epican) (Extracellular matrix receptor III) (ECMR-III) (GP90 lymphocyte homing/adhesion receptor) (HUTCH-I) (Heparan sulfate proteoglycan) (Hermes antigen) (Hyaluronate receptor) (Phagocytic glycoprotein 1) (PGP-1) (Phagocytic glycoprotein I) (PGP-I) (CD antigen CD44) |  |
| P20020 | 0.339877391 | | pH6 | | P20020 | 0.19954 | pH7 | | 1.703304786 | AT2B1_HUMAN | Plasma membrane calcium-transporting ATPase 1 (PMCA1) (EC 3.6.3.8) (Plasma membrane calcium ATPase isoform 1) (Plasma membrane calcium pump isoform 1) |  |
| P08238 | 0.595269239 | | pH6 | | P08238 | 0.364493 | pH7 | | 1.633145342 | HS90B_HUMAN | Heat shock protein HSP 90-beta (HSP 90) (Heat shock 84 kDa) (HSP 84) (HSP84) |  |
| O94832 | 0.131595836 | | pH6 | | O94832 | 0.080725 | pH7 | | 1.63017987 | MYO1D_HUMAN | Unconventional myosin-Id |  |
| P35625 | 0.282815902 | | pH6 | | P35625 | 0.175947 | pH7 | | 1.607392557 | TIMP3_HUMAN | Metalloproteinase inhibitor 3 (Protein MIG-5) (Tissue inhibitor of metalloproteinases 3) (TIMP-3) |  |
| P05023 | 0.925299592 | | pH6 | | P05023 | 0.598166 | pH7 | | 1.546894299 | AT1A1_HUMAN | Sodium/potassium-transporting ATPase subunit alpha-1 (Na(+)/K(+) ATPase alpha-1 subunit) (EC 3.6.3.9) (Sodium pump subunit alpha-1) |  |
| P14618 | 1.016019624 | | pH6 | | P14618 | 0.657645 | pH7 | | 1.544935639 | KPYM_HUMAN | Pyruvate kinase PKM (EC 2.7.1.40) (Cytosolic thyroid hormone-binding protein) (CTHBP) (Opa-interacting protein 3) (OIP-3) (Pyruvate kinase 2/3) (Pyruvate kinase muscle isozyme) (Thyroid hormone-binding protein 1) (THBP1) (Tumor M2-PK) (p58) |  |
| P02751 | 1.274303065 | | pH6 | | P02751 | 0.833404 | pH7 | | 1.529033192 | FINC_HUMAN | Fibronectin (FN) (Cold-insoluble globulin) (CIG) [Cleaved into |  |
| Q6YHK3 | 0.541953049 | | pH6 | | Q6YHK3 | 0.356342 | pH7 | | 1.520881076 | CD109_HUMAN | CD109 antigen (150 kDa TGF-beta-1-binding protein) (C3 and PZP-like alpha-2-macroglobulin domain-containing protein 7) (Platelet-specific Gov antigen) (p180) (r150) (CD antigen CD109) |  |
| P27105 | 0.736584611 | | pH6 | | P27105 | 0.484429 | pH7 | | 1.520522652 | STOM_HUMAN | Erythrocyte band 7 integral membrane protein (Protein 7.2b) (Stomatin) |  |
| **equally expressed** | | | | |  |  |  | |  |  |  | |
| P54920 | | 0.364897777 | | pH6 | P54920 | 0.2561 | pH7 | | 1.42482525 | SNAA_HUMAN | Alpha-soluble NSF attachment protein (SNAP-alpha) (N-ethylmaleimide-sensitive factor attachment protein alpha) |  |
| P04406 | | 1.395905716 | | pH6 | P04406 | 1.010187 | pH7 | | 1.381828544 | G3P_HUMAN | Glyceraldehyde-3-phosphate dehydrogenase (GAPDH) (EC 1.2.1.12) (Peptidyl-cysteine S-nitrosylase GAPDH) (EC 2.6.99.-) |  |
| O43795 | | 0.242661876 | | pH6 | O43795 | 0.177391 | pH7 | | 1.36794943 | MYO1B_HUMAN | Unconventional myosin-Ib (MYH-1c) (Myosin I alpha) (MMI-alpha) (MMIa) |  |
| P43121 | | 1.331016675 | | pH6 | P43121 | 0.977043 | pH7 | | 1.362290583 | MUC18_HUMAN | Cell surface glycoprotein MUC18 (Cell surface glycoprotein P1H12) (Melanoma cell adhesion molecule) (Melanoma-associated antigen A32) (Melanoma-associated antigen MUC18) (S-endo 1 endothelial-associated antigen) (CD antigen CD146) |  |
| P11142 | | 1.42461013 | | pH6 | P11142 | 1.047708 | pH7 | | 1.359739981 | HSP7C_HUMAN | Heat shock cognate 71 kDa protein (Heat shock 70 kDa protein 8) (Lipopolysaccharide-associated protein 1) (LAP-1) (LPS-associated protein 1) |  |
| P11234 | | 0.604962925 | | pH6 | P11234 | 0.465268 | pH7 | | 1.300246368 | RALB_HUMAN | Ras-related protein Ral-B |  |
| P68032 | | 0.557938651 | | pH6 | P68032 | 0.434467 | pH7 | | 1.284190923 | ACTC_HUMAN | Actin, alpha cardiac muscle 1 (Alpha-cardiac actin) |  |
| P07355 | | 1.180872587 | | pH6 | P07355 | 0.953022 | pH7 | | 1.23908278 | ANXA2_HUMAN | Annexin A2 (Annexin II) (Annexin-2) (Calpactin I heavy chain) (Calpactin-1 heavy chain) (Chromobindin-8) (Lipocortin II) (Placental anticoagulant protein IV) (PAP-IV) (Protein I) (p36) |  |
| P43007 | | 1.40895163 | | pH6 | P43007 | 1.157623 | pH7 | | 1.217107168 | SATT_HUMAN | Neutral amino acid transporter A (Alanine/serine/cysteine/threonine transporter 1) (ASCT-1) (SATT) (Solute carrier family 1 member 4) |  |
| Q13683 | | 0.74692912 | | pH6 | Q13683 | 0.618243 | pH7 | | 1.208148684 | ITA7_HUMAN | Integrin alpha-7 [Cleaved into |  |
| Q969P0 | | 0.843520594 | | pH6 | Q969P0 | 0.698882 | pH7 | | 1.206956596 | IGSF8_HUMAN | Immunoglobulin superfamily member 8 (IgSF8) (CD81 partner 3) (Glu-Trp-Ile EWI motif-containing protein 2) (EWI-2) (Keratinocytes-associated transmembrane protein 4) (KCT-4) (LIR-D1) (Prostaglandin regulatory-like protein) (PGRL) (CD antigen CD316) |  |
| P05388 | | 0.138086074 | | pH6 | P05388 | 0.115915 | pH7 | | 1.191272882 | RLA0_HUMAN | 60S acidic ribosomal protein P0 (60S ribosomal protein L10E) |  |
| Q9BYX7 | | 0.197102777 | | pH6 | Q9BYX7 | 0.165456 | pH7 | | 1.191272882 | ACTBM_HUMAN | Putative beta-actin-like protein 3 (Kappa-actin) (POTE ankyrin domain family member K) |  |
| P05106 | | 0.571738787 | | pH6 | P05106 | 0.48172 | pH7 | | 1.18687053 | ITB3_HUMAN | Integrin beta-3 (Platelet membrane glycoprotein IIIa) (GPIIIa) (CD antigen CD61) |  |
| P35241 | | 0.727954712 | | pH6 | P35241 | 0.621967 | pH7 | | 1.170407301 | RADI_HUMAN | Radixin |  |
| P26038 | | 0.749544402 | | pH6 | P26038 | 0.641069 | pH7 | | 1.169209794 | MOES_HUMAN | Moesin (Membrane-organizing extension spike protein) |  |
| P05556 | | 0.567723681 | | pH6 | P05556 | 0.489553 | pH7 | | 1.15967816 | ITB1_HUMAN | Integrin beta-1 (Fibronectin receptor subunit beta) (Glycoprotein IIa) (GPIIA) (VLA-4 subunit beta) (CD antigen CD29) |  |
| P15311 | | 0.580419115 | | pH6 | P15311 | 0.501406 | pH7 | | 1.157583496 | EZRI_HUMAN | Ezrin (Cytovillin) (Villin-2) (p81) |  |
| P35222 | | 0.532802514 | | pH6 | P35222 | 0.465269 | pH7 | | 1.14515018 | CTNB1_HUMAN | Catenin beta-1 (Beta-catenin) |  |
| P23634 | | 0.36267236 | | pH6 | P23634 | 0.316812 | pH7 | | 1.144756996 | AT2B4_HUMAN | Plasma membrane calcium-transporting ATPase 4 (PMCA4) (EC 3.6.3.8) (Matrix-remodeling-associated protein 1) (Plasma membrane calcium ATPase isoform 4) (Plasma membrane calcium pump isoform 4) |  |
| Q9NPH3 | | 0.293365741 | | pH6 | Q9NPH3 | 0.259517 | pH7 | | 1.130428251 | IL1AP_HUMAN | Interleukin-1 receptor accessory protein (IL-1 receptor accessory protein) (IL-1RAcP) (Interleukin-1 receptor 3) (IL-1R-3) (IL-1R3) |  |
| P27348 | | 0.636436411 | | pH6 | P27348 | 0.564661 | pH7 | | 1.127113201 | 1433T_HUMAN | 14-3-3 protein theta (14-3-3 protein T-cell) (14-3-3 protein tau) (Protein HS1) |  |
| P21589 | | 0.831728306 | | pH6 | P21589 | 0.746006 | pH7 | | 1.114907799 | 5NTD_HUMAN | 5'-nucleotidase (5'-NT) (EC 3.1.3.5) (Ecto-5'-nucleotidase) (CD antigen CD73) |  |
| P0CG48 | | 1.047302465 | | pH6 | P0CG48 | 0.945315 | pH7 | | 1.107886943 | UBC_HUMAN | Polyubiquitin-C [Cleaved into |  |
| P50993 | | 0.233466609 | | pH6 | P50993 | 0.210731 | pH7 | | 1.107886943 | AT1A2_HUMAN | Sodium/potassium-transporting ATPase subunit alpha-2 (Na(+)/K(+) ATPase alpha-2 subunit) (EC 3.6.3.9) (Sodium pump subunit alpha-2) |  |
| P08133 | | 0.54664416 | | pH6 | P08133 | 0.495005 | pH7 | | 1.104321209 | ANXA6_HUMAN | Annexin A6 (67 kDa calelectrin) (Annexin VI) (Annexin-6) (Calphobindin-II) (CPB-II) (Chromobindin-20) (Lipocortin VI) (Protein III) (p68) (p70) |  |
| Q15758 | | 2.082580667 | | pH6 | Q15758 | 1.932478 | pH7 | | 1.077673755 | AAAT_HUMAN | Neutral amino acid transporter B(0) (ATB(0)) (Baboon M7 virus receptor) (RD114/simian type D retrovirus receptor) (Sodium-dependent neutral amino acid transporter type 2) (Solute carrier family 1 member 5) |  |
| P08758 | | 1.311208956 | | pH6 | P08758 | 1.23896 | pH7 | | 1.058314411 | ANXA5_HUMAN | Annexin A5 (Anchorin CII) (Annexin V) (Annexin-5) (Calphobindin I) (CBP-I) (Endonexin II) (Lipocortin V) (Placental anticoagulant protein 4) (PP4) (Placental anticoagulant protein I) (PAP-I) (Thromboplastin inhibitor) (Vascular anticoagulant-alpha) (VAC-alpha) |  |
| Q9H4M9 | | 0.732615597 | | pH6 | Q9H4M9 | 0.693777 | pH7 | | 1.055980933 | EHD1_HUMAN | EH domain-containing protein 1 (PAST homolog 1) (hPAST1) (Testilin) |  |
| P08195 | | 1.654610149 | | pH6 | P08195 | 1.56992 | pH7 | | 1.053945814 | 4F2_HUMAN | 4F2 cell-surface antigen heavy chain (4F2hc) (4F2 heavy chain antigen) (Lymphocyte activation antigen 4F2 large subunit) (Solute carrier family 3 member 2) (CD antigen CD98) |  |
| P60709 | | 0.963276579 | | pH6 | P60709 | 0.928602 | pH7 | | 1.037340708 | ACTB_HUMAN | Actin, cytoplasmic 1 (Beta-actin) [Cleaved into |  |
| P61224 | | 0.90216134 | | pH6 | P61224 | 0.873278 | pH7 | | 1.033075038 | RAP1B_HUMAN | Ras-related protein Rap-1b (GTP-binding protein smg p21B) |  |
| Q04917 | | 0.434613174 | | pH6 | Q04917 | 0.426365 | pH7 | | 1.019346123 | 1433F_HUMAN | 14-3-3 protein eta (Protein AS1) |  |
| P35442 | | 0.581840501 | | pH6 | P35442 | 0.573447 | pH7 | | 1.01463706 | TSP2_HUMAN | Thrombospondin-2 |  |
| P62834 | | 0.809615073 | | pH6 | P62834 | 0.79862 | pH7 | | 1.013767087 | RAP1A_HUMAN | Ras-related protein Rap-1A (C21KG) (G-22K) (GTP-binding protein smg p21A) (Ras-related protein Krev-1) |  |
| P62258 | | 0.526348333 | | pH6 | P62258 | 0.52788 | pH7 | | 0.997099347 | 1433E_HUMAN | 14-3-3 protein epsilon (14-3-3E) |  |
| Q8WTV0 | | 0.460719818 | | pH6 | Q8WTV0 | 0.465694 | pH7 | | 0.98931907 | SCRB1_HUMAN | Scavenger receptor class B member 1 (SRB1) (CD36 and LIMPII analogous 1) (CLA-1) (CD36 antigen-like 1) (Collagen type I receptor, thrombospondin receptor-like 1) (SR-BI) (CD antigen CD36) |  |
| Q8WUM4 | | 0.780641054 | | pH6 | Q8WUM4 | 0.790261 | pH7 | | 0.987827176 | PDC6I_HUMAN | Programmed cell death 6-interacting protein (PDCD6-interacting protein) (ALG-2-interacting protein 1) (ALG-2-interacting protein X) (Hp95) |  |
| P47755 | | 0.690532521 | | pH6 | P47755 | 0.708099 | pH7 | | 0.975192415 | CAZA2_HUMAN | F-actin-capping protein subunit alpha-2 (CapZ alpha-2) |  |
| P63104 | | 0.765424657 | | pH6 | P63104 | 0.789574 | pH7 | | 0.96941458 | 1433Z_HUMAN | 14-3-3 protein zeta/delta (Protein kinase C inhibitor protein 1) (KCIP-1) |  |
| Q99988 | | 1.944595311 | | pH6 | Q99988 | 2.016248 | pH7 | | 0.96446241 | GDF15_HUMAN | Growth/differentiation factor 15 (GDF-15) (Macrophage inhibitory cytokine 1) (MIC-1) (NSAID-activated gene 1 protein) (NAG-1) (NSAID-regulated gene 1 protein) (NRG-1) (Placental TGF-beta) (Placental bone morphogenetic protein) (Prostate differentiation factor) |  |
| P62873 | | 0.966224626 | | pH6 | P62873 | 1.007202 | pH7 | | 0.959316055 | GBB1_HUMAN | Guanine nucleotide-binding protein G(I)/G(S)/G(T) subunit beta-1 (Transducin beta chain 1) |  |
| P53675 | | 0.320196302 | | pH6 | P53675 | 0.334501 | pH7 | | 0.957235533 | CLH2_HUMAN | Clathrin heavy chain 2 (Clathrin heavy chain on chromosome 22) (CLH-22) |  |
| O60488 | | 0.196945295 | | pH6 | O60488 | 0.205987 | pH7 | | 0.956105772 | ACSL4_HUMAN | Long-chain-fatty-acid--CoA ligase 4 (EC 6.2.1.3) (Long-chain acyl-CoA synthetase 4) (LACS 4) |  |
| Q9H0U4 | | 0.593638573 | | pH6 | Q9H0U4 | 0.628644 | pH7 | | 0.944316619 | RAB1B_HUMAN | Ras-related protein Rab-1B |  |
| Q00610 | | 1.468208553 | | pH6 | Q00610 | 1.55953 | pH7 | | 0.941442845 | CLH1_HUMAN | Clathrin heavy chain 1 (Clathrin heavy chain on chromosome 17) (CLH-17) |  |
| Q92542 | | 0.316947698 | | pH6 | Q92542 | 0.338518 | pH7 | | 0.936278973 | NICA_HUMAN | Nicastrin |  |
| Q9HAV0 | | 0.385422394 | | pH6 | Q9HAV0 | 0.41175 | pH7 | | 0.936058489 | GBB4_HUMAN | Guanine nucleotide-binding protein subunit beta-4 (Transducin beta chain 4) |  |
| P62879 | | 0.689648025 | | pH6 | P62879 | 0.750194 | pH7 | | 0.91929294 | GBB2_HUMAN | Guanine nucleotide-binding protein G(I)/G(S)/G(T) subunit beta-2 (G protein subunit beta-2) (Transducin beta chain 2) |  |
| O15400 | | 0.685536492 | | pH6 | O15400 | 0.746242 | pH7 | | 0.918652066 | STX7_HUMAN | Syntaxin-7 |  |
| P26006 | | 1.254954368 | | pH6 | P26006 | 1.370089 | pH7 | | 0.915965485 | ITA3_HUMAN | Integrin alpha-3 (CD49 antigen-like family member C) (FRP-2) (Galactoprotein B3) (GAPB3) (VLA-3 subunit alpha) (CD antigen CD49c) [Cleaved into |  |
| P62820 | | 0.476179086 | | pH6 | P62820 | 0.523039 | pH7 | | 0.910408928 | RAB1A_HUMAN | Ras-related protein Rab-1A (YPT1-related protein) |  |
| O00560 | | 0.977031246 | | pH6 | O00560 | 1.074012 | pH7 | | 0.909702459 | SDCB1_HUMAN | Syntenin-1 (Melanoma differentiation-associated protein 9) (MDA-9) (Pro-TGF-alpha cytoplasmic domain-interacting protein 18) (TACIP18) (Scaffold protein Pbp1) (Syndecan-binding protein 1) |  |
| P08582 | | 0.58895541 | | pH6 | P08582 | 0.659836 | pH7 | | 0.892578171 | TRFM_HUMAN | Melanotransferrin (Melanoma-associated antigen p97) (CD antigen CD228) |  |
| P07195 | | 0.218851984 | | pH6 | P07195 | 0.248012 | pH7 | | 0.882424127 | LDHB_HUMAN | L-lactate dehydrogenase B chain (LDH-B) (EC 1.1.1.27) (LDH heart subunit) (LDH-H) (Renal carcinoma antigen NY-REN-46) |  |
| P01023 | | 0.424136643 | | pH6 | P01023 | 0.481615 | pH7 | | 0.880654378 | A2MG_HUMAN | Alpha-2-macroglobulin (Alpha-2-M) (C3 and PZP-like alpha-2-macroglobulin domain-containing protein 5) |  |
| P51149 | | 1.053563384 | | pH6 | P51149 | 1.204769 | pH7 | | 0.87449422 | RAB7A_HUMAN | Ras-related protein Rab-7a |  |
| P36873 | | 0.460719818 | | pH6 | P36873 | 0.537656 | pH7 | | 0.856904885 | PP1G_HUMAN | Serine/threonine-protein phosphatase PP1-gamma catalytic subunit (PP-1G) (EC 3.1.3.16) (Protein phosphatase 1C catalytic subunit) |  |
| Q9P2B2 | | 0.796707426 | | pH6 | Q9P2B2 | 0.936337 | pH7 | | 0.850876807 | FPRP_HUMAN | Prostaglandin F2 receptor negative regulator (CD9 partner 1) (CD9P-1) (Glu-Trp-Ile EWI motif-containing protein F) (EWI-F) (Prostaglandin F2-alpha receptor regulatory protein) (Prostaglandin F2-alpha receptor-associated protein) (CD antigen CD315) |  |
| Q9P265 | | 0.393595965 | | pH6 | Q9P265 | 0.462599 | pH7 | | 0.850836504 | DIP2B_HUMAN | Disco-interacting protein 2 homolog B (DIP2 homolog B) |  |
| P06756 | | 9.543489748 | | pH6 | P06756 | 11.51214 | pH7 | | 0.828993197 | ITAV_HUMAN | Integrin alpha-V (Vitronectin receptor subunit alpha) (CD antigen CD51) [Cleaved into |  |
| P61204 | | 0.607090442 | | pH6 | P61204 | 0.742465 | pH7 | | 0.817668988 | ARF3_HUMAN | ADP-ribosylation factor 3 |  |
| P31946 | | 0.780959342 | | pH6 | P31946 | 0.968531 | pH7 | | 0.806333559 | 1433B_HUMAN | 14-3-3 protein beta/alpha (Protein 1054) (Protein kinase C inhibitor protein 1) (KCIP-1) [Cleaved into |  |
| O14672 | | 0.311207699 | | pH6 | O14672 | 0.395622 | pH7 | | 0.786629799 | ADA10_HUMAN | Disintegrin and metalloproteinase domain-containing protein 10 (ADAM 10) (EC 3.4.24.81) (CDw156) (Kuzbanian protein homolog) (Mammalian disintegrin-metalloprotease) (CD antigen CD156c) |  |
| Q96BY6 | | 0.06144556 | | pH6 | Q96BY6 | 0.079501 | pH7 | | 0.772890046 | DOC10_HUMAN | Dedicator of cytokinesis protein 10 (Zizimin-3) |  |
| Q9BZQ8 | | 0.352493416 | | pH6 | Q9BZQ8 | 0.464702 | pH7 | | 0.758536584 | NIBAN_HUMAN | Protein Niban (Cell growth-inhibiting gene 39 protein) (Protein FAM129A) |  |
| P61981 | | 0.577166468 | | pH6 | P61981 | 0.79574 | pH7 | | 0.725320602 | 1433G_HUMAN | 14-3-3 protein gamma (Protein kinase C inhibitor protein 1) (KCIP-1) [Cleaved into |  |
| Q9BQE3 | | 0.882822248 | | pH6 | Q9BQE3 | 1.232738 | pH7 | | 0.716147541 | TBA1C_HUMAN | Tubulin alpha-1C chain (Alpha-tubulin 6) (Tubulin alpha-6 chain) [Cleaved into |  |
| Q9HD67 | | 0.451169558 | | pH6 | Q9HD67 | 0.630947 | pH7 | | 0.715067732 | MYO10_HUMAN | Unconventional myosin-X (Unconventional myosin-10) |  |
| P68363 | | 0.999385025 | | pH6 | P68363 | 1.405317 | pH7 | | 0.711145416 | TBA1B_HUMAN | Tubulin alpha-1B chain (Alpha-tubulin ubiquitous) (Tubulin K-alpha-1) (Tubulin alpha-ubiquitous chain) [Cleaved into |  |
| Q9Y696 | | 0.496008548 | | pH6 | Q9Y696 | 0.698683 | pH7 | | 0.709919171 | CLIC4_HUMAN | Chloride intracellular channel protein 4 (Intracellular chloride ion channel protein p64H1) |  |
| P50395 | | 0.186021088 | | pH6 | P50395 | 0.267424 | pH7 | | 0.695603813 | GDIB_HUMAN | Rab GDP dissociation inhibitor beta (Rab GDI beta) (Guanosine diphosphate dissociation inhibitor 2) (GDI-2) |  |
| Q7L576 | | 0.213309742 | | pH6 | Q7L576 | 0.309611 | pH7 | | 0.688961361 | CYFP1_HUMAN | Cytoplasmic FMR1-interacting protein 1 (Specifically Rac1-associated protein 1) (Sra-1) (p140sra-1) |  |
| Q9Y376 | | 0.313911745 | | pH6 | Q9Y376 | 0.479822 | pH7 | | 0.654225848 | CAB39_HUMAN | Calcium-binding protein 39 (MO25alpha) (Protein Mo25) |  |
| P20742 | | 0.087505107 | | pH6 | P20742 | 0.139961 | pH7 | | 0.625212589 | PZP_HUMAN | Pregnancy zone protein (C3 and PZP-like alpha-2-macroglobulin domain-containing protein 6) |  |
| O75054 | | 0.407431399 | | pH6 | O75054 | 0.663688 | pH7 | | 0.613889848 | IGSF3_HUMAN | Immunoglobulin superfamily member 3 (IgSF3) (Glu-Trp-Ile EWI motif-containing protein 3) (EWI-3) |  |
| O75083 | | 0.239376378 | | pH6 | O75083 | 0.393435 | pH7 | | 0.60842633 | WDR1_HUMAN | WD repeat-containing protein 1 (Actin-interacting protein 1) (AIP1) (NORI-1) |  |
| P54709 | | 0.422940976 | | pH6 | P54709 | 0.712163 | pH7 | | 0.593882139 | AT1B3_HUMAN | Sodium/potassium-transporting ATPase subunit beta-3 (Sodium/potassium-dependent ATPase subunit beta-3) (ATPB-3) (CD antigen CD298) |  |
| P35221 | | 0.233266147 | | pH6 | P35221 | 0.408752 | pH7 | | 0.570678624 | CTNA1_HUMAN | Catenin alpha-1 (Alpha E-catenin) (Cadherin-associated protein) (Renal carcinoma antigen NY-REN-13) |  |
| P07814 | | 0.044818112 | | pH6 | P07814 | 0.086187 | pH7 | | 0.520012269 | SYEP_HUMAN | Bifunctional glutamate/proline--tRNA ligase (Bifunctional aminoacyl-tRNA synthetase) (Cell proliferation-inducing gene 32 protein) (Glutamatyl-prolyl-tRNA synthetase) [Includes |  |
| **pH6 down-regulated** | | | | |  |  |  | |  |  |  | |
| P61026 | | 0.221293721 | | pH6 | P61026 | 0.455915 | pH7 | | 0.485384067 | RAB10_HUMAN | Ras-related protein Rab-10 |  |
| P07437 | | 0.563592643 | | pH6 | P07437 | 1.270703 | pH7 | | 0.443528335 | TBB5_HUMAN | Tubulin beta chain (Tubulin beta-5 chain) |  |
| P52907 | | 0.35489282 | | pH6 | P52907 | 0.813819 | pH7 | | 0.436083221 | CAZA1_HUMAN | F-actin-capping protein subunit alpha-1 (CapZ alpha-1) |  |
| P37802 | | 0.224150298 | | pH6 | P37802 | 0.538106 | pH7 | | 0.416553977 | TAGL2_HUMAN | Transgelin-2 (Epididymis tissue protein Li 7e) (SM22-alpha homolog) |  |
| P60033 | | 0.533742135 | | pH6 | P60033 | 1.313951 | pH7 | | 0.406211493 | CD81_HUMAN | CD81 antigen (26 kDa cell surface protein TAPA-1) (Target of the antiproliferative antibody 1) (Tetraspanin-28) (Tspan-28) (CD antigen CD81) |  |
| Q14108 | | 0.212154648 | | pH6 | Q14108 | 0.534767 | pH7 | | 0.396723193 | SCRB2_HUMAN | Lysosome membrane protein 2 (85 kDa lysosomal membrane sialoglycoprotein) (LGP85) (CD36 antigen-like 2) (Lysosome membrane protein II) (LIMP II) (Scavenger receptor class B member 2) (CD antigen CD36) |  |
| Q08431 | | 1.208190341 | | pH6 | Q08431 | 3.168296 | pH7 | | 0.381337556 | MFGM_HUMAN | Lactadherin (Breast epithelial antigen BA46) (HMFG) (MFGM) (Milk fat globule-EGF factor 8) (MFG-E8) (SED1) [Cleaved into |  |
| P08107 | | 0.144107238 | | pH6 | P08107 | 0.429009 | pH7 | | 0.335906938 | HS71A_HUMAN | Heat shock 70 kDa protein 1A (Heat shock 70 kDa protein 1) (HSP70-1) (HSP70.1) |  |
| P09543 | | 0.126100698 | | pH6 | P09543 | 0.38296 | pH7 | | 0.329278608 | CN37_HUMAN | 2',3'-cyclic-nucleotide 3'-phosphodiesterase (CNP) (CNPase) (EC 3.1.4.37) |  |
| P01892 | | 0.186010823 | | pH6 | P01892 | 0.628653 | pH7 | | 0.295887847 | 1A02_HUMAN | HLA class I histocompatibility antigen, A-2 alpha chain (MHC class I antigen A*2) |  |
| Q13509 | | 0.274026769 | | pH6 | Q13509 | 0.945733 | pH7 | | 0.289750783 | TBB3_HUMAN | Tubulin beta-3 chain (Tubulin beta-4 chain) (Tubulin beta-III) |  |
| Q99536 | | 0.176692704 | | pH6 | Q99536 | 0.630111 | pH7 | | 0.280415125 | VAT1_HUMAN | Synaptic vesicle membrane protein VAT-1 homolog (EC 1.-.-.-) |  |
| P68371 | | 0.339416231 | | pH6 | P68371 | 1.213316 | pH7 | | 0.279742548 | TBB4B_HUMAN | Tubulin beta-4B chain (Tubulin beta-2 chain) (Tubulin beta-2C chain) |  |
| P04350 | | 0.279824251 | | pH6 | P04350 | 1.123329 | pH7 | | 0.249102649 | TBB4A_HUMAN | Tubulin beta-4A chain (Tubulin 5 beta) (Tubulin beta-4 chain) |  |
| Q8WWI5 | | 0.084882726 | | pH6 | Q8WWI5 | 0.367104 | pH7 | | 0.231222844 | CTL1_HUMAN | Choline transporter-like protein 1 (CDw92) (Solute carrier family 44 member 1) (CD antigen CD92) |  |
| P04899 | | 0.176692704 | | pH6 | P04899 | 0.831302 | pH7 | | 0.212549411 | GNAI2_HUMAN | Guanine nucleotide-binding protein G(i) subunit alpha-2 (Adenylate cyclase-inhibiting G alpha protein) |  |
|  | |  | |  | O14817 | 0.660787 | pH7 | | pH7 specific | TSN4_HUMAN | Tetraspanin-4 (Tspan-4) (Novel antigen 2) (NAG-2) (Transmembrane 4 superfamily member 7) |  |
|  | |  | |  | O14908 | 0.397694 | pH7 | | pH7 specific | GIPC1_HUMAN | PDZ domain-containing protein GIPC1 (GAIP C-terminus-interacting protein) (RGS-GAIP-interacting protein) (RGS19-interacting protein 1) (Synectin) (Tax interaction protein 2) (TIP-2) |  |
|  | |  | |  | O15013 | 0.110345 | pH7 | | pH7 specific | ARHGA_HUMAN | Rho guanine nucleotide exchange factor 10 |  |
|  | |  | |  | O43491 | 0.092643 | pH7 | | pH7 specific | E41L2_HUMAN | Band 4.1-like protein 2 (Generally expressed protein 4.1) (4.1G) |  |
|  | |  | |  | O94804 | 0.107818 | pH7 | | pH7 specific | STK10_HUMAN | Serine/threonine-protein kinase 10 (EC 2.7.11.1) (Lymphocyte-oriented kinase) |  |
|  | |  | |  | P00338 | 0.09346 | pH7 | | pH7 specific | LDHA_HUMAN | L-lactate dehydrogenase A chain (LDH-A) (EC 1.1.1.27) (Cell proliferation-inducing gene 19 protein) (LDH muscle subunit) (LDH-M) (Renal carcinoma antigen NY-REN-59) |  |
|  | |  | |  | P06744 | 0.177447 | pH7 | | pH7 specific | G6PI_HUMAN | Glucose-6-phosphate isomerase (GPI) (EC 5.3.1.9) (Autocrine motility factor) (AMF) (Neuroleukin) (NLK) (Phosphoglucose isomerase) (PGI) (Phosphohexose isomerase) (PHI) (Sperm antigen 36) (SA-36) |  |
|  | |  | |  | P08581 | 0.144755 | pH7 | | pH7 specific | MET_HUMAN | Hepatocyte growth factor receptor (HGF receptor) (EC 2.7.10.1) (HGF/SF receptor) (Proto-oncogene c-Met) (Scatter factor receptor) (SF receptor) (Tyrosine-protein kinase Met) |  |
|  | |  | |  | P08754 | 0.516299 | pH7 | | pH7 specific | GNAI3_HUMAN | Guanine nucleotide-binding protein G(k) subunit alpha (G(i) alpha-3) |  |
|  | |  | |  | P09211 | 0.466849 | pH7 | | pH7 specific | GSTP1_HUMAN | Glutathione S-transferase P (EC 2.5.1.18) (GST class-pi) (GSTP1-1) |  |
|  | |  | |  | P09471 | 0.435331 | pH7 | | pH7 specific | GNAO_HUMAN | Guanine nucleotide-binding protein G(o) subunit alpha |  |
|  | |  | |  | P13591 | 0.142055 | pH7 | | pH7 specific | NCAM1_HUMAN | Neural cell adhesion molecule 1 (N-CAM-1) (NCAM-1) (CD antigen CD56) |  |
|  | |  | |  | P13929 | 0.149485 | pH7 | | pH7 specific | ENOB_HUMAN | Beta-enolase (EC 4.2.1.11) (2-phospho-D-glycerate hydro-lyase) (Enolase 3) (Muscle-specific enolase) (MSE) (Skeletal muscle enolase) |  |
|  | |  | |  | P15880 | 0.154977 | pH7 | | pH7 specific | RS2_HUMAN | 40S ribosomal protein S2 (40S ribosomal protein S4) (Protein LLRep3) (Small ribosomal subunit protein uS5) |  |
|  | |  | |  | P17813 | 0.493291 | pH7 | | pH7 specific | EGLN_HUMAN | Endoglin (CD antigen CD105) |  |
|  | |  | |  | P20340 | 0.265824 | pH7 | | pH7 specific | RAB6A_HUMAN | Ras-related protein Rab-6A (Rab-6) |  |
|  | |  | |  | P23471 | 0.117017 | pH7 | | pH7 specific | PTPRZ_HUMAN | Receptor-type tyrosine-protein phosphatase zeta (R-PTP-zeta) (EC 3.1.3.48) (Protein-tyrosine phosphatase receptor type Z polypeptide 1) (Protein-tyrosine phosphatase receptor type Z polypeptide 2) (R-PTP-zeta-2) |  |
|  | |  | |  | P27701 | 1.603516 | pH7 | | pH7 specific | CD82_HUMAN | CD82 antigen (C33 antigen) (IA4) (Inducible membrane protein R2) (Metastasis suppressor Kangai-1) (Suppressor of tumorigenicity 6 protein) (Tetraspanin-27) (Tspan-27) (CD antigen CD82) |  |
|  | |  | |  | P30050 | 0.239111 | pH7 | | pH7 specific | RL12_HUMAN | 60S ribosomal protein L12 (Large ribosomal subunit protein uL11) |  |
|  | |  | |  | P30443 | 0.840616 | pH7 | | pH7 specific | 1A01_HUMAN | HLA class I histocompatibility antigen, A-1 alpha chain (MHC class I antigen A*1) |  |
|  | |  | |  | P31150 | 0.117552 | pH7 | | pH7 specific | GDIA_HUMAN | Rab GDP dissociation inhibitor alpha (Rab GDI alpha) (Guanosine diphosphate dissociation inhibitor 1) (GDI-1) (Oligophrenin-2) (Protein XAP-4) |  |
|  | |  | |  | P35613 | 0.716982 | pH7 | | pH7 specific | BASI_HUMAN | Basigin (5F7) (Collagenase stimulatory factor) (Extracellular matrix metalloproteinase inducer) (EMMPRIN) (Leukocyte activation antigen M6) (OK blood group antigen) (Tumor cell-derived collagenase stimulatory factor) (TCSF) (CD antigen CD147) |  |
|  | |  | |  | P40925 | 0.091912 | pH7 | | pH7 specific | MDHC_HUMAN | Malate dehydrogenase, cytoplasmic (EC 1.1.1.37) (Cytosolic malate dehydrogenase) (Diiodophenylpyruvate reductase) (EC 1.1.1.96) |  |
|  | |  | |  | P49006 | 1.299081 | pH7 | | pH7 specific | MRP_HUMAN | MARCKS-related protein (MARCKS-like protein 1) (Macrophage myristoylated alanine-rich C kinase substrate) (Mac-MARCKS) (MacMARCKS) |  |
|  | |  | |  | P51148 | 0.237643 | pH7 | | pH7 specific | RAB5C_HUMAN | Ras-related protein Rab-5C (L1880) (RAB5L) |  |
|  | |  | |  | P62491 | 0.249873 | pH7 | | pH7 specific | RB11A_HUMAN | Ras-related protein Rab-11A (Rab-11) (YL8) |  |
|  | |  | |  | P63092 | 0.324827 | pH7 | | pH7 specific | GNAS2_HUMAN | Guanine nucleotide-binding protein G(s) subunit alpha isoforms short (Adenylate cyclase-stimulating G alpha protein) |  |
|  | |  | |  | Q14344 | 0.26852 | pH7 | | pH7 specific | GNA13_HUMAN | Guanine nucleotide-binding protein subunit alpha-13 (G alpha-13) (G-protein subunit alpha-13) |  |
|  | |  | |  | Q562R1 | 0.098435 | pH7 | | pH7 specific | ACTBL_HUMAN | Beta-actin-like protein 2 (Kappa-actin) |  |
|  | |  | |  | Q9NZH0 | 0.811764 | pH7 | | pH7 specific | GPC5B_HUMAN | G-protein coupled receptor family C group 5 member B (A-69G12.1) (Retinoic acid-induced gene 2 protein) (RAIG-2) |  |
|  | |  | |  | Q9UKS6 | 0.288199 | pH7 | | pH7 specific | PACN3_HUMAN | Protein kinase C and casein kinase substrate in neurons protein 3 (SH3 domain-containing protein 6511) |  |
|  | |  | |  | Q9Y6E0 | 0.695213 | pH7 | | pH7 specific | STK24_HUMAN | Serine/threonine-protein kinase 24 (EC 2.7.11.1) (Mammalian STE20-like protein kinase 3) (MST-3) (STE20-like kinase MST3) [Cleaved into: Serine/threonine-protein kinase 24 36 kDa subunit (Mammalian STE20-like protein kinase 3 N-terminal) (MST3/N) |  |
