# Supplementary material for: Acidic microenvironment plays a key role in human melanoma progression through a sustained exosome mediated transfer of clinically relevant metastatic molecules
Source: J Exp Clin Cancer Res. 2018 Oct 5;37:245. doi: 10.1186/s13046-018-0915-z (PMC6173926; doi:10.1186/s13046-018-0915-z)
Supplement: Supplementary file 12 — Table S3. pH 6.0 upregulated categories involved in metastatic processes are indicated. Proteins found in these categories are listed. (DOCX 15 kb) [file 13046_2018_915_MOESM12_ESM.docx]

**Table S3**. pH 6.0 upregulated categories involved in metastatic processes are indicated.

| **Regulation of actin cytoskeleton** | | | |  |  |  |  |
| --- | --- | --- | --- | --- | --- | --- | --- |
| P01112 | HRas proto-oncogene, GTPase(HRAS) | | | | | | |
| P46940 | IQ motif containing GTPase activating protein 1(IQGAP1) | | | | | | |
| P12814 | actinin alpha 1(ACTN1) | | | | | | |
| O43707 | actinin alpha 4(ACTN4) | | | | | | |
| P60953 | cell division cycle 42(CDC42) | | | | | | |
| P23528 | cofilin 1(CFL1) | | | | | | |
| Q9Y281 | cofilin 2(CFL2) | | | | | | |
| P02751 | fibronectin 1(FN1) | | | | | | |
| P06396 | gelsolin(GSN) | | | | | | |
| P01111 | neuroblastoma RAS viral oncogene homolog(NRAS) | | | | | | |
| P18206 | vinculin(VCL) | | | | | | |
|  |  |  |  |  |  |  |  |
| **Proteoglycans in cancer** | | |  |  |  |  |  |
| P16070 | CD44 molecule (Indian blood group)(CD44) | | | | | | |
| P01112 | HRas proto-oncogene, GTPase(HRAS) | | | | | | |
| P46940 | IQ motif containing GTPase activating protein 1(IQGAP1) | | | | | | |
| P35625 | TIMP metallopeptidase inhibitor 3(TIMP3) | | | | | | |
| P60953 | cell division cycle 42(CDC42) | | | | | | |
| P02751 | fibronectin 1(FN1) | | | | | | |
| P01111 | neuroblastoma RAS viral oncogene homolog(NRAS) | | | | | | |
| P07996 | thrombospondin 1(THBS1) | | | | | | |
|  |  |  |  |  |  |  |  |
| **Focal adhesion** | |  |  |  |  |  |  |
| P01112 | HRas proto-oncogene, GTPase(HRAS) | | | | | | |
| P12814 | actinin alpha 1(ACTN1) | | | | | | |
| O43707 | actinin alpha 4(ACTN4) | | | | | | |
| P60953 | cell division cycle 42(CDC42) | | | | | | |
| P02751 | fibronectin 1(FN1) | | | | | | |
| Q9Y490 | talin 1(TLN1) | | | | | | |
| P07996 | thrombospondin 1(THBS1) | | | | | | |
| P18206 | vinculin(VCL) | | | | | | |
|  |  |  |  |  |  |  |  |
| **Leukocyte transendhothelial migration** | | | | |  |  |  |
| P12814 | actinin alpha 1(ACTN1) | | | | | | |
| O43707 | actinin alpha 4(ACTN4) | | | | | | |
| O60716 | catenin delta 1(CTNND1) | | | | | | |
| P60953 | cell division cycle 42(CDC42) | | | | | | |
| P05362 | intercellular adhesion molecule 1(ICAM1) | | | | | | |
| P18206 | vinculin(VCL) | | | | | | |
|  |  |  |  |  |  |  |  |
| **Protein processing in endoplasmic reticulum** | | | | |  |  |  |
| O60884 | DnaJ heat shock protein family (Hsp40) member A2(DNAJA2) | | | | | | |
| Q14697 | glucosidase II alpha subunit(GANAB) | | | | | | |
| P07900 | heat shock protein 90 alpha family class A member 1(HSP90AA1) | | | | | | |
| P08238 | heat shock protein 90 alpha family class B member 1(HSP90AB1) | | | | | | |
| P14625 | heat shock protein 90 beta family member 1(HSP90B1) | | | | | | |
| P34931 | heat shock protein family A (Hsp70) member 1 like(HSPA1L) | | | | | | |
| P11021 | heat shock protein family A (Hsp70) member 5(HSPA5) | | | | | | |
| Q9Y4L1 | hypoxia up-regulated 1(HYOU1) | | | | | | |
| P55072 | valosin containing protein(VCP) | | | | | | |
