# Supplementary material for: Acidic microenvironment plays a key role in human melanoma progression through a sustained exosome mediated transfer of clinically relevant metastatic molecules
Source: J Exp Clin Cancer Res. 2018 Oct 5;37:245. doi: 10.1186/s13046-018-0915-z (PMC6173926; doi:10.1186/s13046-018-0915-z)
Supplement: Supplementary file 14 — Additional References. (DOCX 17 kb) [file 13046_2018_915_MOESM14_ESM.docx]

**Additional References**

1. Colombo MP, Maccalli C, Mattei S, Melani C, Radrizzani M, Parmiani G. Expression of cytokine genes, including IL-6, in human malignant melanoma cell lines. Melanoma Res. 1992;2:181-9.
2. Liu XY, Lai F, Yan XG, Jiang CC, Guo ST, Wang CY, et al. RIP Kinase Is an Oncogenic Driver in Melanoma. Cancer Res. 2015;75:1736-48.
3. Hoek K, Rimm DL, Williams KR, Zhao H, Ariyan S, Lin A, et al. Expression profiling reveals novel pathways in the transformation of melanocytes to melanomas. Cancer Res. 2004;64:5270-82.
4. Marincola FM, Hijazi YM, Fetsch P, Salgaller ML, Rivoltini L, Cormier J, et al. Analysis of expression of the melanoma-associated antigens MART-1 and gp100 in metastatic melanoma cell lines and in in situ lesions. J Immunother Emphasis Tumor Immunol. 1996;19:192-205.
5. Bellenghi M, Puglisi R, Pedini F, De Feo A, Felicetti F, Bottero L, et al. SCD5-induced oleic acid production reduces melanoma malignancy by intracellular retention of SPARC and cathepsin B. J Pathol. 2015;236:315-25.
6. Monzani E, Facchetti F, Galmozzi E, Corsini E, Benetti A, Cavazzin C, et al. Melanoma contains CD133 and ABCG2 positive cells with enhanced tumourigenic potential. Eur J Cancer. 2007;43:935-46.
7. Zimmerer RM, Korn P, Demougin P, Kampmann A, Kokemüller H, Eckardt AM, et al. Functional features of cancer stem cells in melanoma cell lines. Cancer Cell Int. 2013;13:78.
8. Smalley KS, Contractor R, Nguyen TK, Xiao M, Edwards R, Muthusamy V, et al. Identification of a novel subgroup of melanomas with KIT/cyclin-dependent kinase-4 overexpression. Cancer Res. 2008;68:5743-52.
9. Shields JM, Thomas NE, Cregger M, Berger AJ, Leslie M, Torrice C, et al. Lack of extracellular signal-regulated kinase mitogen-activated protein kinase signaling shows a new type of melanoma. Cancer Res. 2007;67:1502-12.
10. Klijn C, Durinck S, Stawiski EW, Haverty PM, Jiang Z, Liu H, et al. A comprehensive transcriptional portrait of human cancer cell lines. Nat Biotechnol. 2015;33:306-12.
11. James-Kracke M.R. Quick and accurate method to convert BCECF fluorescence to pHi: calibration in three different types of cell preparations. J Cell Physiol. 1992 Jun;151:596-603.
